# Transcriptional profiling reveals changes in gene regulation and signaling transduction pathways during temperature stress in wucai (*Brassica campestris* L.)

**DOI:** 10.1186/s12864-021-07981-9

**Published:** 2021-09-22

**Authors:** Lingyun Yuan, Yushan Zheng, Libing Nie, Liting Zhang, Ying Wu, Shidong Zhu, Jinfeng Hou, Guo Lei Shan, Tong Kun Liu, Guohu Chen, Xiaoyan Tang, Chenggang Wang

**Affiliations:** 1 College of Horticulture, Vegetable Genetics and Breeding Laboratory, Anhui Agricultural University, 130 West Changjiang Road, Hefei, 230036 Anhui China; 2 Provincial Engineering Laboratory for Horticultural Crop Breeding of Anhui, 130 West of Changjiang Road, Hefei, 230036 Anhui China; 3 Wanjiang Vegetable Industrial Technology Institute, Maanshan, 238200 Anhui China; 4 College of Horticulture, Nanjing Agricultural University, Nanjing, 210095 China

**Keywords:** Wucai, Temperature stress, RNA-Seq, Differentially expressed genes, Photosynthesis, *BrLhc* superfamily

## Abstract

**Background:**

Wucai (*Brassica campestris* L. ssp. *chinensis* var. *rosularis* Tsen) is a cold-tolerant plant that is vulnerable to high temperature. This study explored the response mechanism of wucai to low temperature. In this study, wucai seedlings were treated with different temperatures, including low temperature (LT), high temperature (HT), and a control.

**Results:**

According to transcriptomics analysis, the number of differentially expressed genes (DEGs) in HT and LT was 10,702 and 7267, respectively, compared with the control. The key genes associated with the physiological response of wucai to the treatments were analyzed. The Kyoto Encyclopedia of Genes and Genomes and Gene Ontology annotations indicated the importance of the photosynthesis and photosynthetic-antenna protein pathways. We found that a high-temperature environment greatly inhibited the expression of important genes in the photosynthetic pathway (*BrLhc* superfamily members, *PsaD, PsaE, PsaD, PsaD, PsbO, PsbP, PsbQ, PsbR, PsbS, PsbW, PsbY, Psb27,* and *Psb28*), whereas low temperature resulted in the expression of certain key genes (*BrLhc* superfamily members, *Psa F, Psa H, Psb S, Psb H, Psb 28*). In addition, the wucai seedlings exhibited better photosynthetic performance under low-temperature conditions than high-temperature conditions.

**Conclusions:**

Based on the above results, we speculate that upon exposure to low temperature, the plants developed higher cold tolerance by upregulating the expression of genes related to photosynthesis. Conversely, high-temperature stress inhibited the expression of pivotal genes and weakened the self-regulating ability of the plants.

**Supplementary Information:**

The online version contains supplementary material available at 10.1186/s12864-021-07981-9.

## Background

Temperature stress is major abiotic stress that influences the survival, geographical distribution, and yield of plants on a global scale. To manage thermal stress, plants modify their physiology, morphology, metabolic pathways, and cellular and sub-cellular structures via signal transduction and the expression regulation of genes related to temperature stress [1–3].

Under heat stress, the balance between reactive oxygen species (ROS) production and elimination is rapidly disrupted, leading to an increase in ROS content. Heat stress may also lead to the increased production of reactive nitrogen species (RNS), which have toxic effects on cells, resulting in nitrosative stress [4–6]. Cold stress can cause damage to a plant by reducing photosynthetic rates, which arises from the direct inhibition of metabolic enzymes by cold stress and the reprogramming of gene expression [7, 8]. Both cold and heat stress can change the fluidity of cellular phospholipid membranes [9]. This type of change can be sensed by integral membrane proteins, allowing for stress transcription factors to activate stress-responsive genes [10]. Finally, these pathways lead to adjustments in plant metabolism and development that are aimed at reaching homeostasis under stressful conditions [11, 12].

In addition to the physiological responses to cold and heat, plant resistance to stress is often activated by alterations in gene expression. Transcriptomics is used to evaluate systematic changes in gene expression and estimate the response of plants to temperature stress. The positive roles of the high expression of *COR* genes and the conserved mechanism of circadian clock-related genes in the response of tobacco to cold stress provided some valuable genes for crop improvement under cold stress [13]. The transcriptome of *Chlamydomonas reinhardtii* exposed to cold indicated differentially expressed genes (DEGs) that were associated with protein synthesis, cell cycle, and protein kinase-based phosphorylation [14]. The BcHSP70 gene obtained from *Brassica campestris* was transferred into tobacco. After high temperature treatment, it was found that compared with wild-type tobacco, the chlorophyll content in the transformant was increased, the SOD and POD activities were improved, the specific conductivity and MDA content were decreased, the accumulation of proline and soluble sugar was increased [15]. Under heat stress, the expression of HSP70 in cabbage increased by about 3 times, and HSP70 can be used as an index to identify the heat tolerance of cabbage [16]. *Arabidopsis* CAMTA1, CAMTA2 and CAMTA3 promote low temperature and freezing tolerance by activating the CBF (C-repeat/DRE binding factor) transcription factor [17]. ICE1 is a well-defined helix-loop-helix (bHLH) protein, which can be used as an upstream regulator of the cold response transcriptional regulation cascade in *Arabidopsis*. ICE1 regulates the transcriptional expression of downstream genes by binding to the MYC element (CANNTG) on the CBF gene promoter, and CBF regulates many cold regulatory (COR) genes [18–20]. In wheat, according to transcriptome profiling, Hsp-family, ascorbate peroxidase, β-amylase, γ-gliadin-2, and LMW-glutenin were upregulated under heat stress in the developing grains [21]. Song et al. (2016) analyzed cold and heat treatments in non-heading Chinese cabbage using RNA sequencing (RNA-Seq). The enrichment analyses identified 33 DEGs and 25 DEGs under heat and cold treatment, respectively [22].

In our previous study, tandem mass tag (TMT) labeling was used to analyze the changes between HT-treated and LT-treated plants at the protein level [23]. The results indicated that 172 differentially expressed proteins exhibited simultaneous upregulation in response to both HT and LT and were related to photosynthesis, carbohydrate metabolism, redox homeostasis, chaperones, heat-shock proteins, and signal transduction pathways. Wucai grows under low-temperature environments, such as late autumn or winter in the Yangtze-Huaihe River Basin. Once wucai is exposed to a high-temperature environment, its growth will be inhibited and heat damage will occur [24]. Elucidating the regulatory pattern of the response of wucai to LT is thus of great interest. The wucai genotype ‘WS-1’, a variety of non-heading Chinese cabbage with higher tolerance to LT than other germplasms, was selected in this study to illustrate the physiological and biochemical aspects involved in the response to both LT from the aspects of morphology, photosynthetic function, and related gene transcription level alterations. A perspective on the specific low temperature tolerance mechanism of wucai, as a hardy crop, is also presented.

## Results

### The effect of different temperature stress treatments on the growth parameters of wucai seedlings

**Fig. 1:**
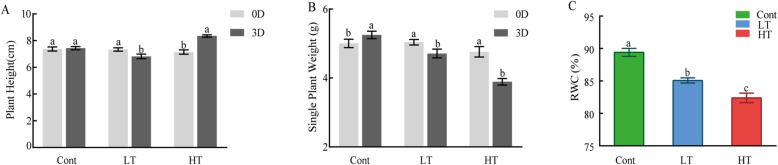
The effects of different temperature stresses on the plant height (**a**), single plant weight (**b**), and relative water content (RWC, **c**) of the leaves of wucai. Values represent the mean ± SE (*n* = 3). Letters indicate significant differences at *P* < 0.05 according to Duncan’s multiple range tests. Representative data from five independent experiments are shown

After 3d of HT treatment, the leaves of wucai turned yellow and curled significantly. After 3d of LT treatment, there was no obvious change in the appearance of the seedlings. As shown in the figure (Additional file: Figure S1), the growth status of wucai seedlings changed differently after 3d of treatment at different temperatures. After high temperature treatment, the plant height was significantly higher than that before treatment, while after low temperature treatment, the plant height was significantly lower than that before treatment, but there was no significant change in the control group at room temperature for 3 days. The single plant weight after treatment also showed different changes (Fig. 1a). The single plant weight after high temperature and low temperature treatment was significantly decreased by 18.28 and 6.50%, respectively, while it was significantly increased by 4.93% in the normal temperature control group (Fig. 1b). RWC of LT and HT were significantly lower than Cont, with the relative water content of LT being higher than that of HT (Fig. 1c).

### The effect of different temperature stress treatments on membrane lipid oxidation damage

**Fig. 2:**
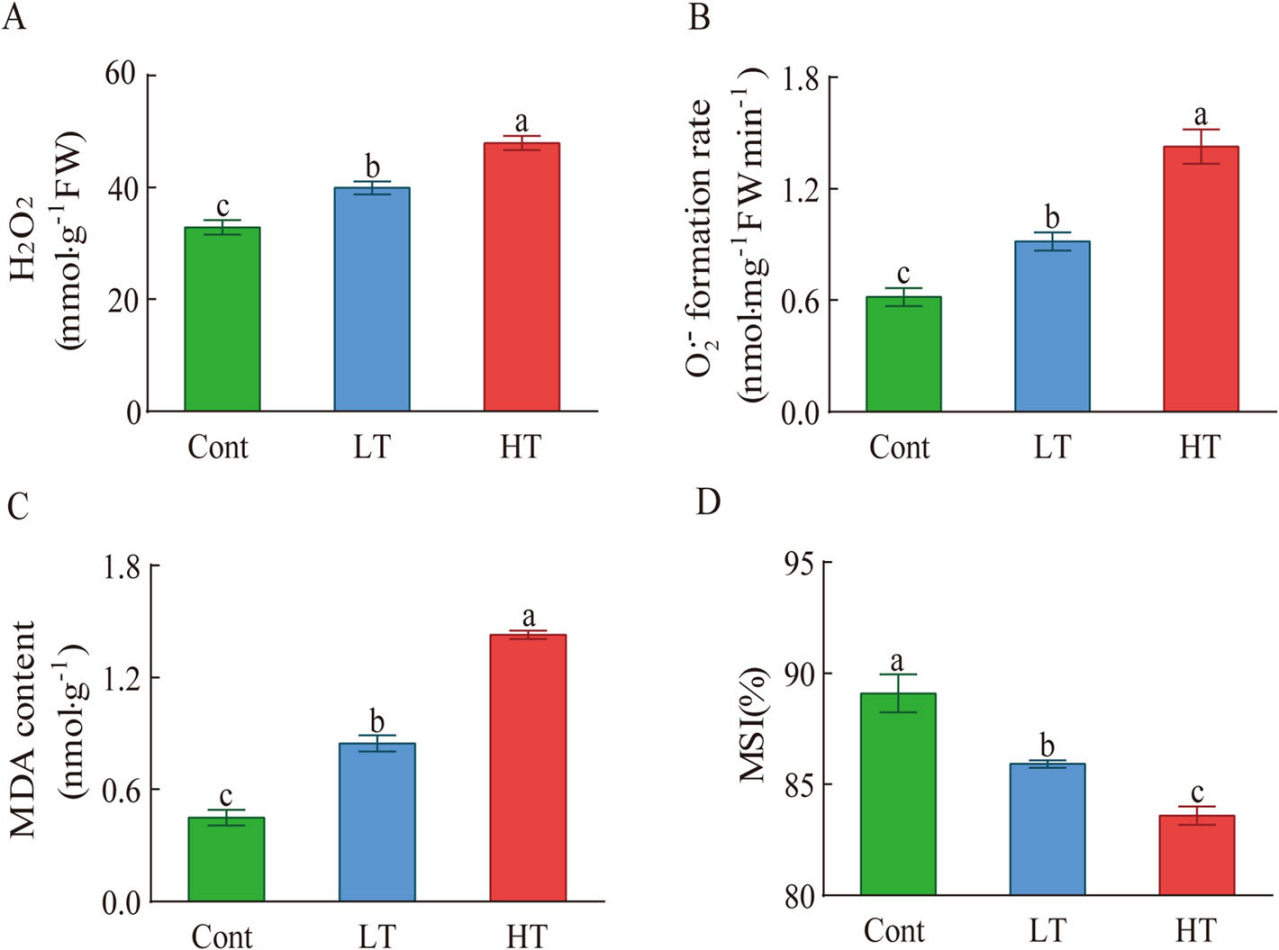
The effects of different temperature stresses on the H_2_O_2_ content (**a**), O_2_^•-^ formation rate (**b**), MDA content (**c**), MSI (**d**), Values represent the mean ± SE (*n* = 3). Letters indicate significant differences at *P* < 0.05 according to Duncan’s multiple range tests

The physiological parameters of the wucai seedlings treated under different temperatures showed different trends. The malondialdehyde content (MDA) indicates the degree of lipid peroxidation in the plant cell membrane. The accumulation of MDA will break the integrity of the membrane and reduce the MSI. Compared with Cont, both LT and HT had a higher MDA content, and the MDA content of HT was obviously higher than that of LT. The MDA contents of LT and HT were 89.04 and 219.14% higher than Cont, respectively (Fig. 2c). The membrane stability index (MSI) showed a downward trend after the two temperature stress treatments, and the degree of decline in HT was significantly greater than that in LT (Fig. 2d). In addition, the accumulation of H_2_O_2_ and O_2_^•-^ in seedlings leads to membrane lipid peroxidation. For the same treatment over 3 d, the H_2_O_2_ content and O_2_^•-^ formation rate of the experimental materials under LT were significantly lower than under HT (Fig. 2a, b).

### Transcriptome profiles

**Fig. 3:**
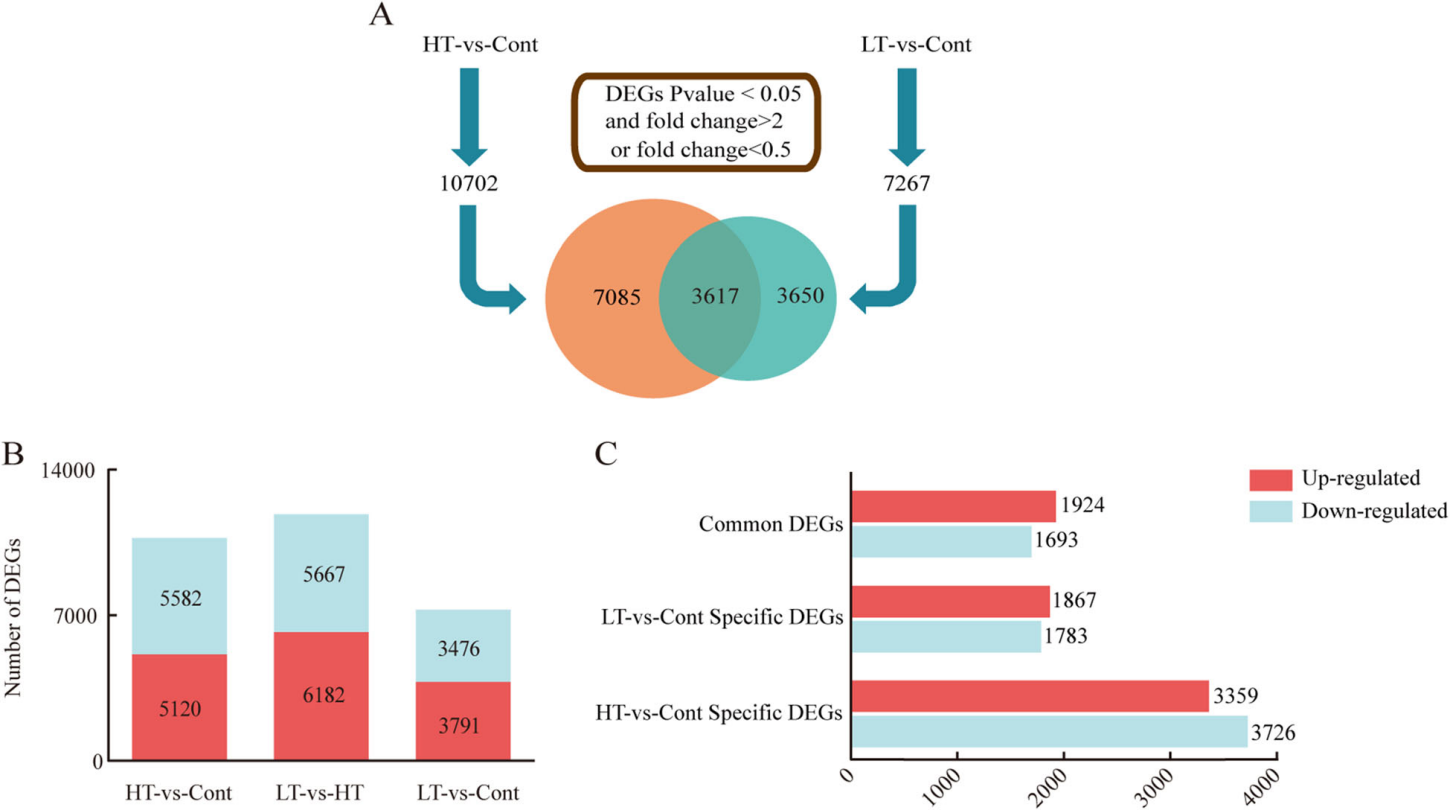
Differential gene expression in response to temperature stress under low temperature (LT) and high temperature (HT) treatments. **a** The number of DEGs in the leaves of wucai under LT-vs-Cont and HT-vs-Cont. The value shared by the two ellipses indicates the number of genes that are co-regulated. **b** Expression of DEGs in HT-vs-Cont, LT-vs-HT, LT-vs-Cont. **c** Common DEG for both LT-vs-Cont and HT-vs-Cont, including the distribution of DEGs specifically regulated by each. *P* value < 0.05 and foldChange > 2 or foldChange < 0.5 was set as the threshold for significantly differential expression

Nine samples were sequenced and mapped to the reference transcriptome, the reference genome for this transcriptome sequencing comparison is the genome of the NCBI database: GCF_000309985.1 (Brapa_1.0), and a total of 62.97 G of clean data were obtained. The effective data volume of each sample was 6.04–8.01 G, the Q30 scores were 94.35–95.17%, and the average GC content was 47.74% (Additional file: Table S1). Screening was conducted based on the expression level of the DEGs in the different groups. There were two different groups in total, and compared with the control, the numbers of DEGs detected under HT-vs-Cont and LT-vs-Cont conditions were 10,702 and 7267, respectively (Fig. 3a). Hierarchical clustering of all of the DEGs was conducted to observe the gene expression patterns and was evaluated by the log10RPKMs of the 2 groups. Among the 7267 DEGs in LT-vs-Cont, 3791 genes were upregulated and 3476 were downregulated. In the LT-vs-Cont group, there were 5120 upregulated genes and 5582 downregulated genes (Fig. 3b, Additional file: Figure S2). These DEGs were divided into three groups: (1) 3650 DEGs that were specific to the LT-vs-Cont group, including 1783 downregulated genes, and 1867 upregulated genes, (2) DEGs that were common to both the LT-vs-Cont and HT-vs-Cont groups, including 3617 common genes, 1693 common downregulated genes, and 1924 common upregulated genes, (3) and 7085 DEGs that were specific to HT-vs-Cont, including 3726 downregulated genes, and 3359 upregulated genes (Fig. 3a, c). To further study the potential mechanism of WS-1 as a cold-resistant vegetable that is adapted to low temperatures.

### GO and KEGG analysis of the DEGs of LT and HT

GO enrichment analysis was used to further explore the functions of DEGs at different temperatures (Additional file: Figure S3) The results revealed the distribution of the 3605 DEGs in the LT-vs-Cont and 7085 DEGs in the HT-vs-Cont in the three ontology types. The transcriptome analysis sifted GO terms, according to the corresponding the number of differential genes of each term; 10 terms were sorted from large to small. In the HT-vs-Cont, response to salt stress, regulation of transcription, DNA-templated, and response to abscisic acid were the most enriched pathways, whereas in the LT-vs-Cont group, the DEGs were mostly associated with translation, response to salt stress, and response to cold in the biological process category. In the cellular component category, the HT-vs-Cont enriched DEGs were mainly related to nucleus, integral component of membrane, and cytoplasm, whereas in LT-vs-Cont, these included nucleus, chloroplast, and cytosol. In the molecular function category in HT-vs-Cont, ATP binding was the most abundant, followed by metal ion binding, and DNA-binding transcription factor activity, while in LT-vs-Cont, structural constituent of ATP binding, structural constituent of ribosome, and metal ion binding.

**Fig. 4:**
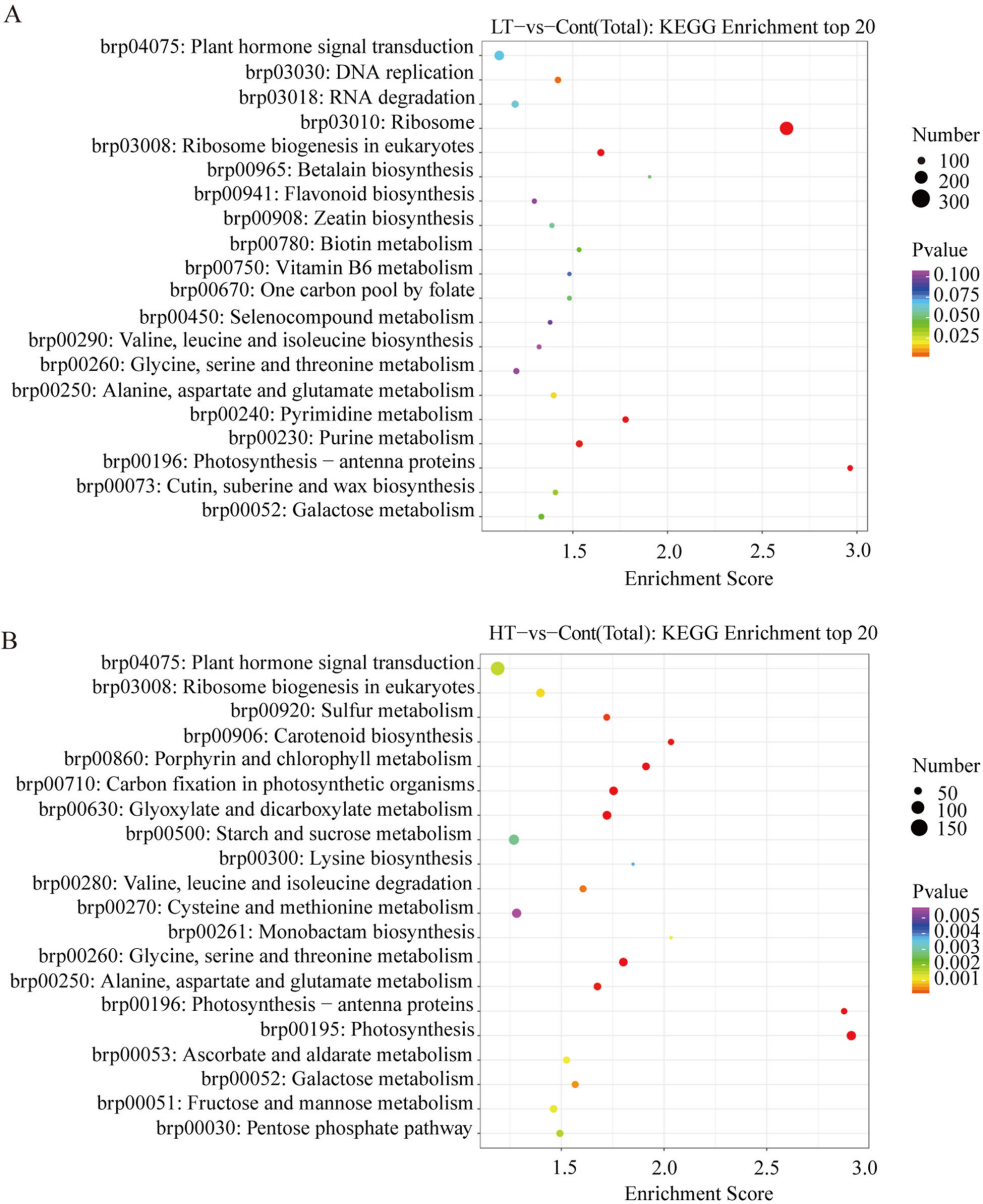
KEGG enrichment analysis results, with the 20 most enriched KEGG terms shown. High and low *P*-values are represented by blue and red, respectively. KEGG pathway enrichment analyses of the DEGs in the LT-vs-Cont (**a**). KEGG pathway enrichment analyses of the DEGs in the HT-vs-Cont (**b**)

The enriched KEGG pathways of the DEGs differed between the different temperature treatments. In LT-vs-Cont, there were 10 pathway annotations that were significantly enriched, there are mainly pathway: ribosome, photosynthesis-antenna proteins, ribosome biogenesis in eukaryotes, pyrimidine metabolism, and purine metabolism (Fig. 4a). In HT-vs-Cont, there were 10 pathway annotations that were significantly enriched, the mainly pathway including photosynthesis, photosynthesis-antenna proteins, glycine, serine, and threonine metabolism, carbon fixation in photosynthetic organisms, and glyoxylate and dicarboxylate metabolism (Fig. 4b).

### Effect of temperature stress on photosynthesis pathway analysis

**Fig. 5:**
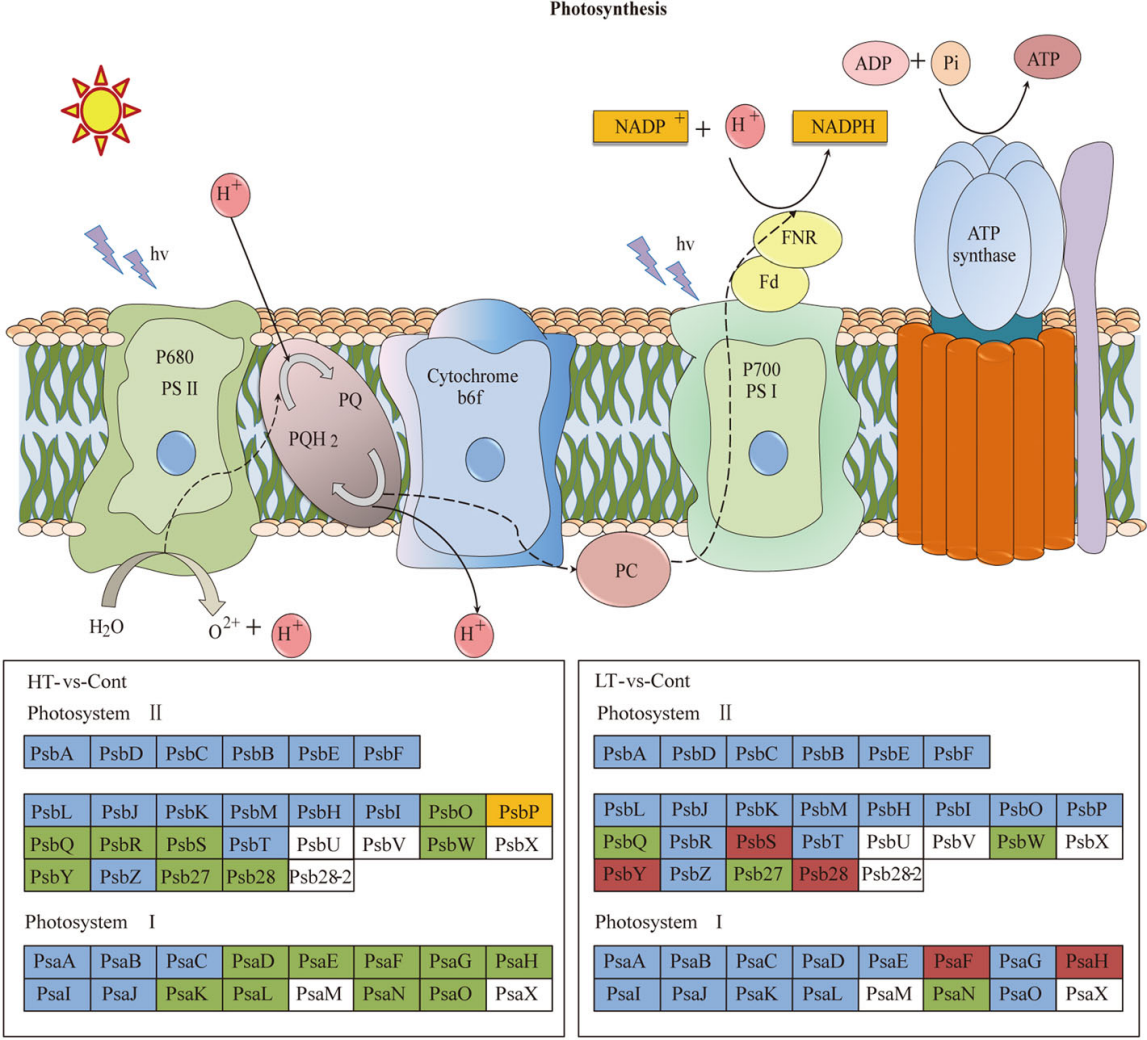
Photosynthesis-related gene expression in wucai leaves under LT and HT based on the KEGG pathway analysis. Red, green, and blue represent upregulated, downregulated, and nonregulated genes

Following low temperature treatment, some genes in the photosynthesis pathway were upregulated. In PSI, subunit III (*PsaF*) and subunit VI (*PsaH*) were upregulated. In PSII, thylakoid photoprotective gene (*PsbS*), *Psb H,* and *Psb 28* were upregulated. Overall, 8 genes were upregulated and 5 genes were downregulated. Following high temperature treatment, some genes in the photosynthesis pathway were downregulated in PSI, including *PsaD, PsaE, PsaD, PsaD, in* PSII*,* including *PsbO, PsbP, PsbQ, PsbR, PsbS, PsbW, PsbY, Psb27,* and *Psb28*. Overall, there were 4 genes with upregulated expression and 71 genes with downregulated expression (Fig. 5, Additional file: Figure S4, Tables S2, S3).

### The effect of different temperature stress treatments on the Chl content of the wucai seedlings

**Fig. 6:**
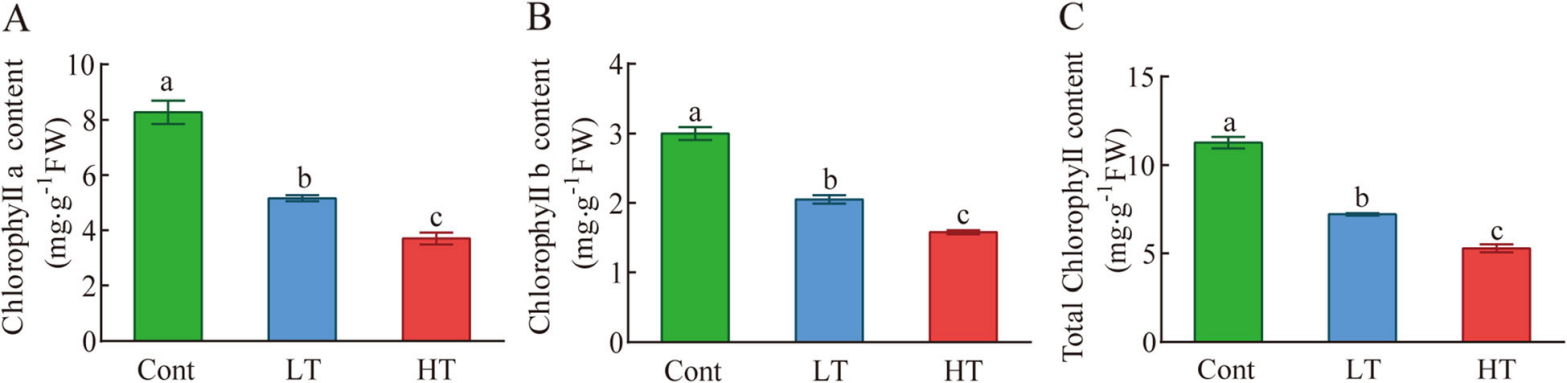
Effects of photosynthetic pigment contents in wucai leaves under temperature stress. **A–C** Quantification of the Chl *a* content (a), Chl *b* content (b), total Chl content (c). The data are presented as the mean ± SE. Bars with different letters above the columns indicate significant differences (*P* < 0.05, Duncan’s range test) on a given day of treatment

To gain some insights into the different effects of the different temperature stresses on the growth process of wucai seedlings, we measured the contents of Chl *a*, Chl *b*, and total Chl (Fig. 6). We found that temperature stress caused chlorophyll degradation. The Chl *a* content, Chl *b* content, and total Chl content of LT and HT were lower than Cont. However, the above indicators were significantly higher under LT than HT.

**Fig. 7:**
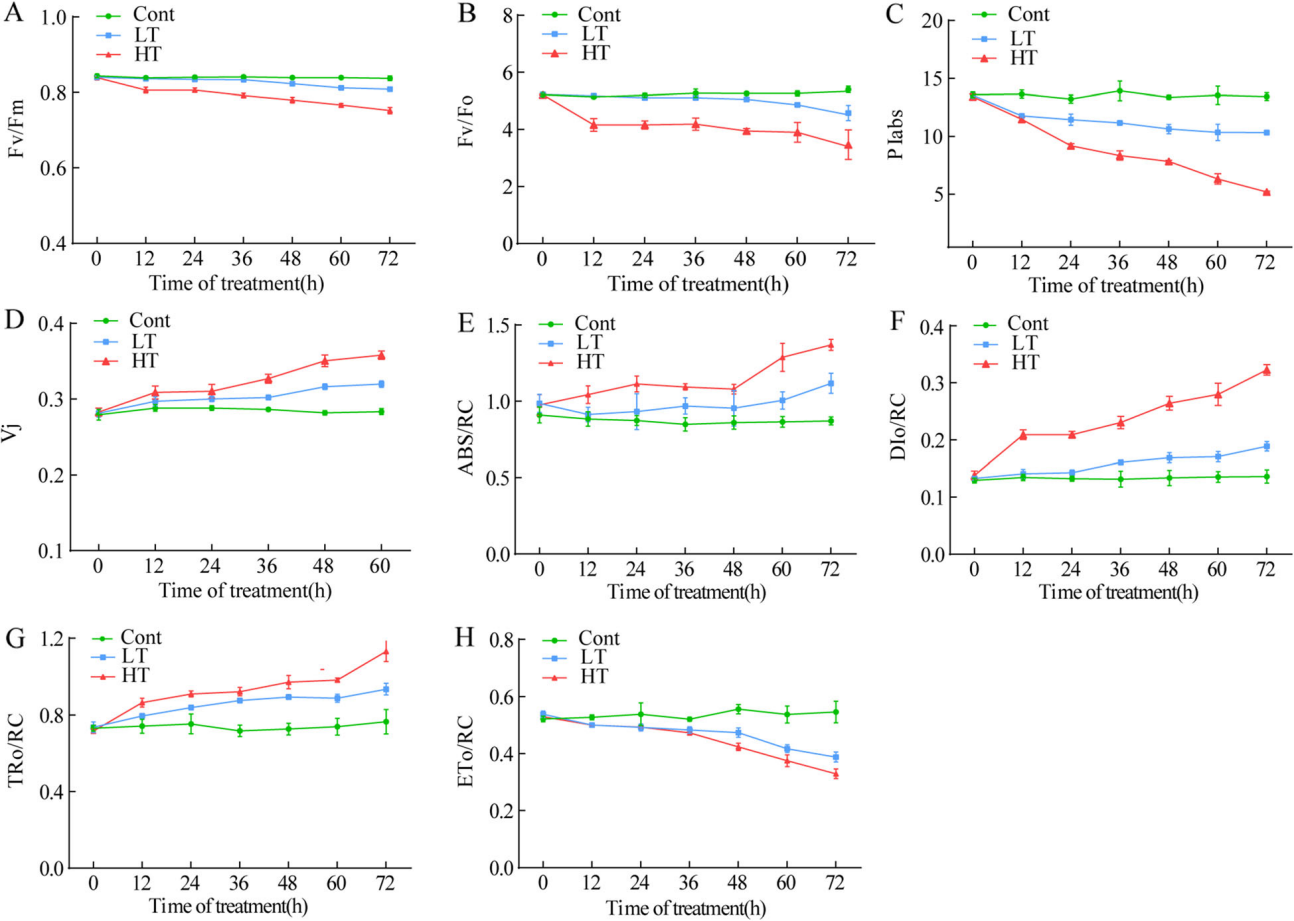
The influence of different temperature stresses on F_v_/F_m_ (**a**), F_v_/F_o_ (**b**), PI_abs_ (**c**), V_j_ (**d**), ABS/RC(**e**), DI_O_/RC(**f**), TR_O_/RC(**g**), and ET_O_/RC(**h**). The data are presented as the mean ± SE. Representative data from five independent experiments are shown

The F_v_/F_m_, F_v_/F_o_, PI_abs_, and V_j_ of the wucai seedlings under a normal growth state exhibited a stable trend over 3 d, with no significant changes observed. The F_v_/F_m_, F_v_/F_o_, and PI_abs_ of the wucai seedlings showed a downward trend during the 3 d of low temperature and high temperature treatments and were reduced on the third day of treatment. Compared with before the treatment, Fv/Fm of LT and HT decreased by 3.73 and 10.37%, respectively, and the F_v_/F_o_ dropped by 13.55 and 34.75%, respectively. PI_abs_ decreased by 23.82 and 61.42%, respectively (Fig. 7a, b, c). After the different temperature treatments, V_j_ gradually increased. It can be seen from that the increase in HT was more significant than that of LT (Fig. 7d). After 3 days of different temperature stress treatment, ABS/RC, DIO/RC, TRO/RC of wucai seedlings all increased, and the increase was more in wucai seedlings after high temperature treatment, but ETO/RC showed a downward trend, and the decrease was more in wucai seedlings after high temperature treatment (Fig. 7e, f, g, h).

### Effects of different temperature stresses on the gas exchange parameters of the wucai seedlings

**Fig. 8:**
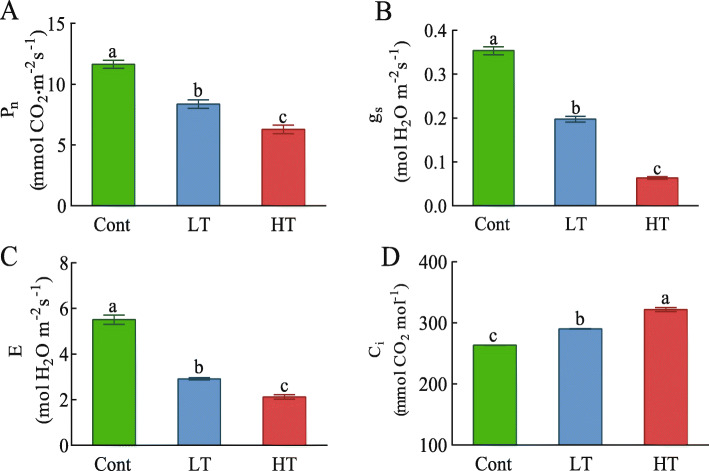
The influence of different temperature stresses on the photosynthetic rate (P_N_, **A**), stomatal conductance (g_s_, **B**), transpiration rate (E, **C**), and intercellular CO_2_ concentration (C_i_, **D**). The data are presented as the mean ± SE. Bars with different letters above the columns indicate significant differences (*P* < 0.05, Duncan’s range test) on a given day of treatment

As shown in the result, different temperature stresses had different effects on the wucai seedlings. The P_N_, g_s_, and E of LT and HT were significantly lower than that of Cont. Compared with Cont, sampled treated with LT decreased by 28.20, 44.17, 47.02%, respectively, whereas sampled treated with HT decreased by 46.03, 82.07, and 61.47%, respectively. On the contrary, under temperature stress, the C_i_ value of the wucai seedlings increased significantly, the effect of HT was higher that the one of LT (Fig. 8).

### Effect of temperature stress on photosynthesis-antenna proteins pathway analysis

**Fig. 9:**
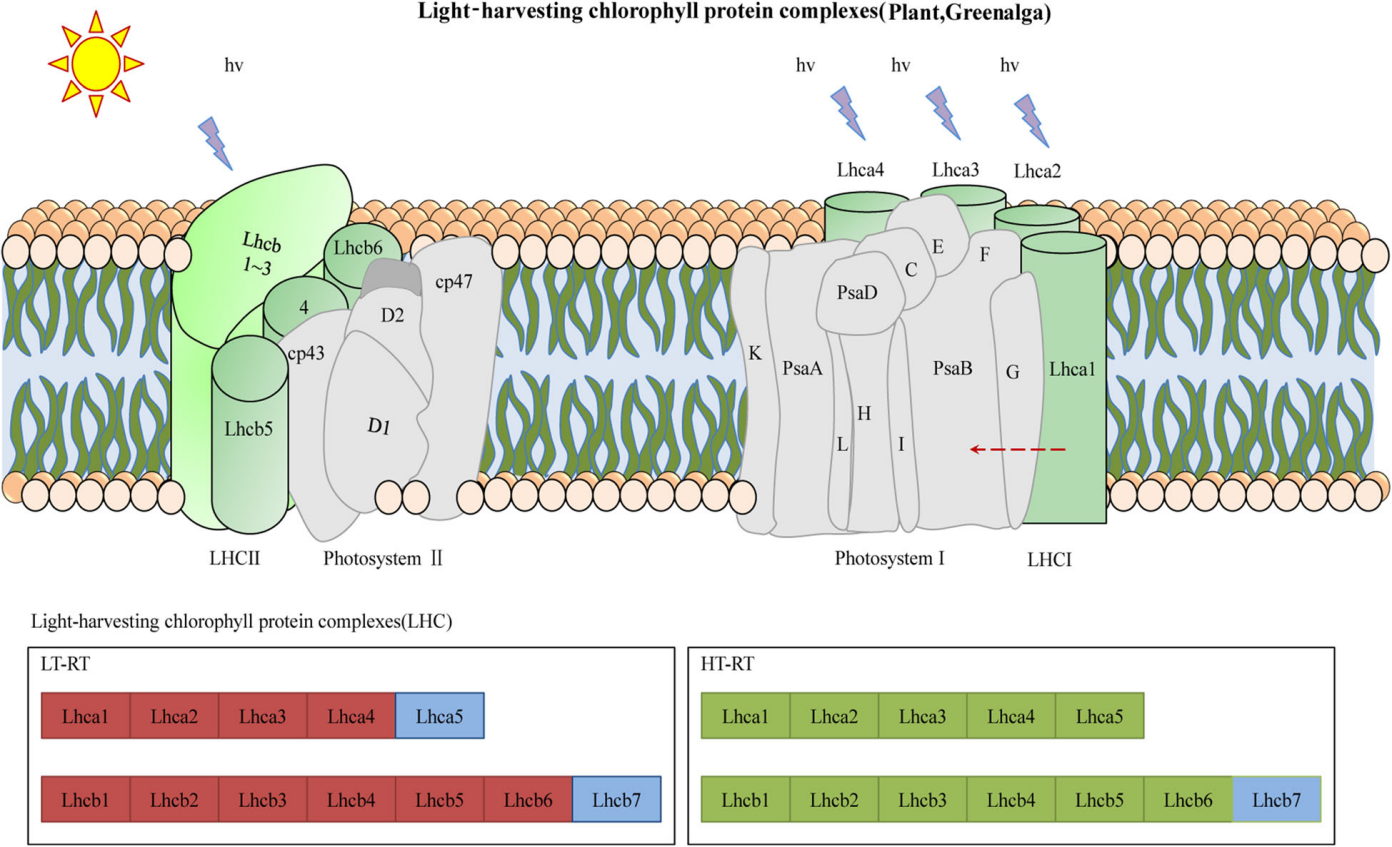
Photosynthesis-antenna proteins-related gene expression in wucai leaves influenced by LT and HT based on the KEGG pathway analysis. Red, green, and blue represent the upregulated, downregulated, and nonregulated genes

In the photosynthesis-antenna protein pathway, the expression of DEGs exhibited opposite trends between the different temperature treatments. Most of these DEGs encoded proteins concentrated in light-harvesting chlorophyll protein complex (LHC). There were 28 downregulated genes in HT-vs-Cont and 22 upregulated genes in LT-vs-Cont (Fig. 9, Additional file: Figure S5, Tables S4, S5).

By searching the *Brassica rapa* genome, 47 members of the *BrLhc* superfamily were identified and named. The physicochemical properties of the members of the *BrLhc* superfamily of wucai are shown in the table (Additional file: Table S6). The longest *BrLhc* protein of wucai contained 496 amino acid residues and the shortest contained 148 residues. The molecular weight (Mw) ranged between 8.43 and 54.93 kDa, and the isoelectric point (pI) ranged between 4.85 and 11.48. the open reading frame (ORF) length was between 231 and 1491, 87.23% of the members were located in chloroplasts, and the others were located in the extracellular space, cytoplasm, nucleus, and plasma membrane.

To better understand the evolutionary relationships and structural diversity of the BrLhc protein, we used MEGA7.0 software to conduct a phylogenetic analysis of the BrLhc protein. The online program MEME Server was used to analyze the conserved motifs of the BrLhc proteins, and the tool TBtools was used to analyze the structure of the *BrLhc* gene online. Almost all *BrLhc* genes had a highly conserved exon-intron organization. The five *BrLhc* genes possessed no introns, and each subfamily contained similar gene structures (Additional file: Figure S6).

**Fig. 10:**
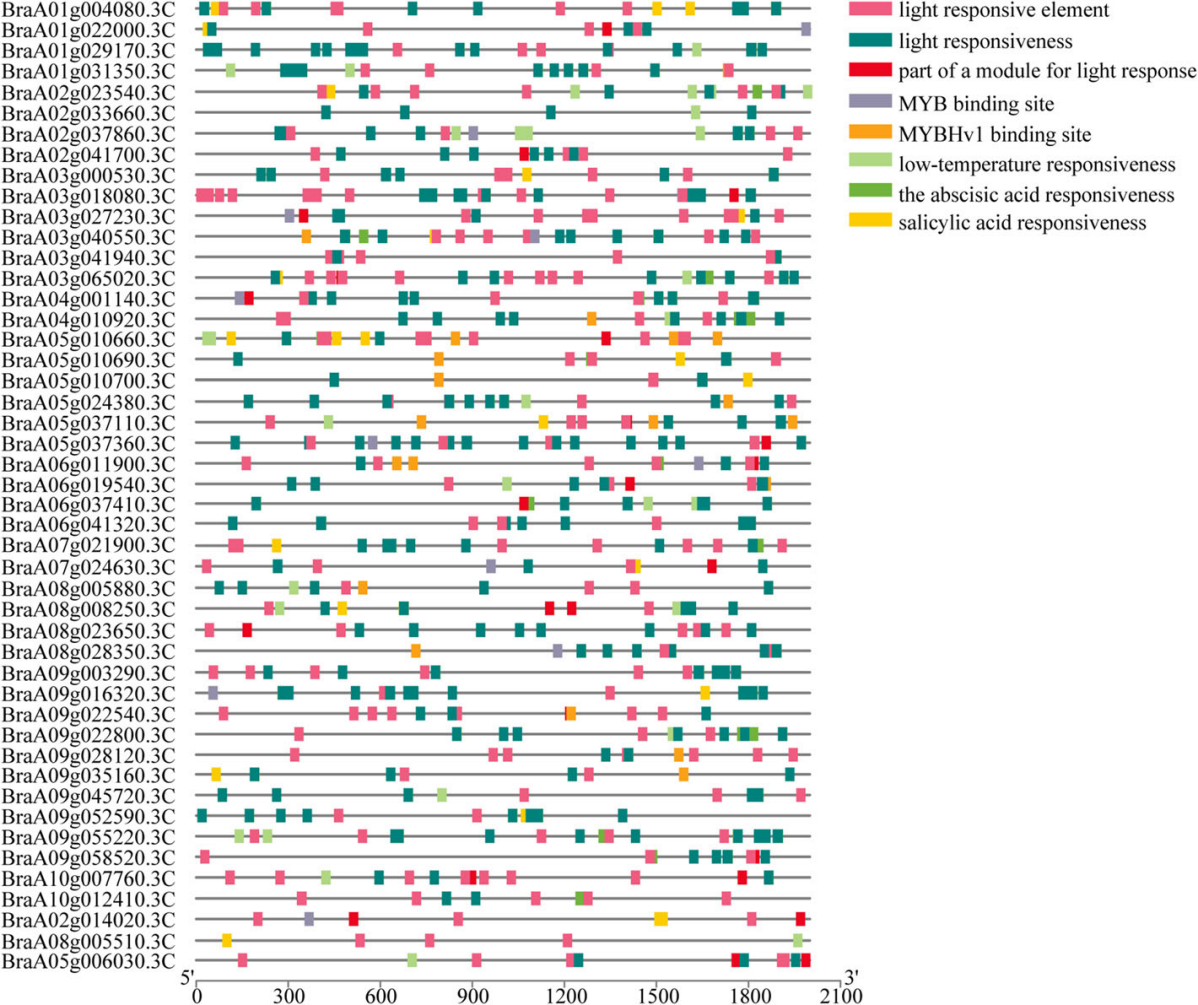
*Cis*-acting elements of the *BrLhc* superfamily. Colored rectangles represent different cis-acting elements

The results of the *cis*-acting elements of the *BrLhc* superfamily genes showed that the regulatory elements were related to light responsiveness, *MYB* binding site, abscisic acid responsiveness, salicylic acid responsiveness, defense and stress responsiveness, low-temperature responsiveness, and *MYBHv1* binding site (Fig. 10).

### Quantitative RT-PCR analysis

**Fig. 11:**
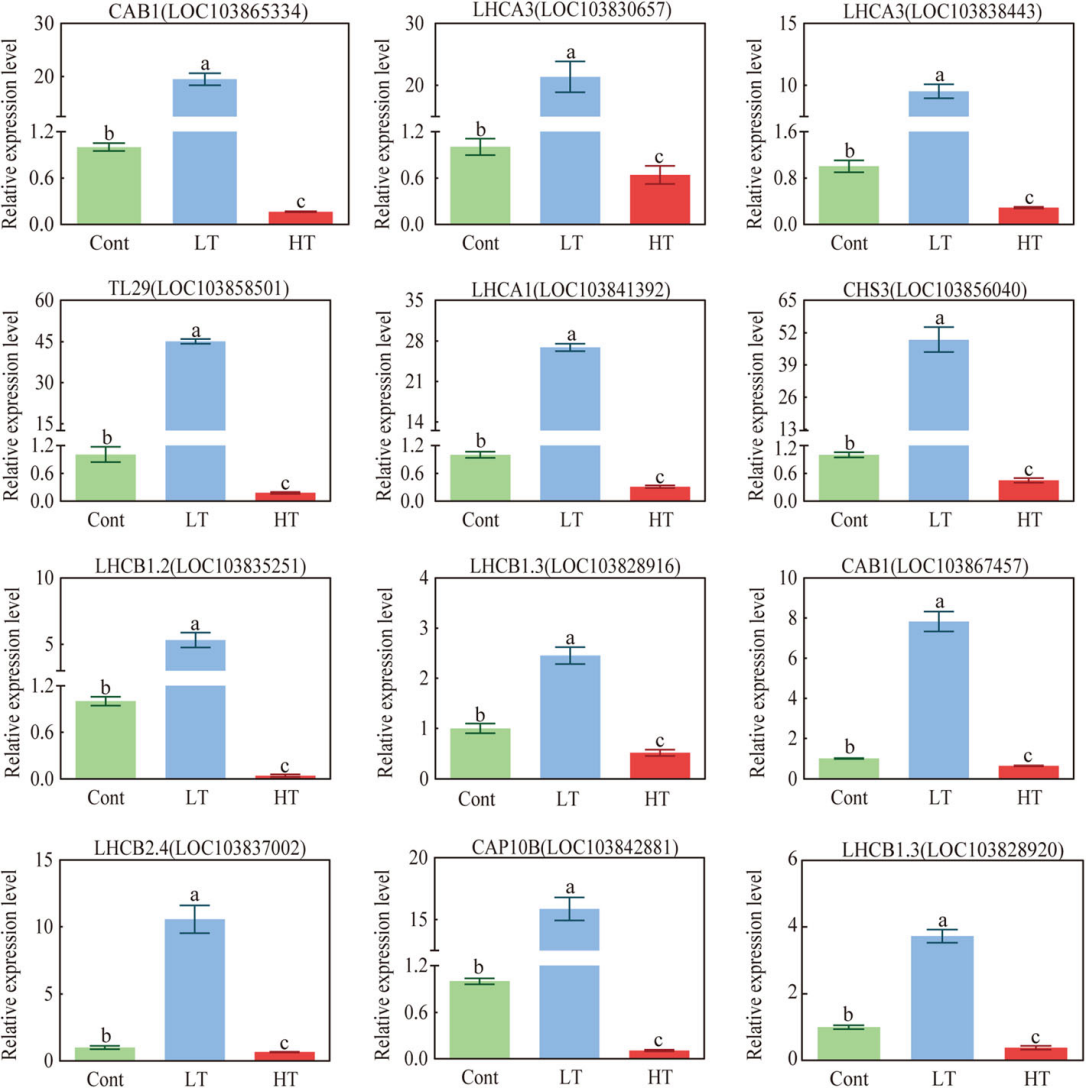
The effects of different temperature stress on photosynthesis-related genes in the leaves of wucai. The data are presented as the mean ± SE. Bars with different letters above the columns indicate significant differences (*P* < 0.05, Duncan’s range test) on a given day of treatment

To confirm the quality of the transcriptome data, 12 DEGs participating in the photosynthesis-antenna protein pathway, glutathione metabolism, and flavonoid biosynthesis were selected for verification by qRT-PCR analysis (Additional file: Table S7). The results showed that the expression of the randomly selected differential genes was consistent with the transcriptome results (Fig. 11).

## Discussion

Wucai is a cold-tolerant vegetable that is not resistant to heat stress. To evaluate the specific temperature stress tolerance mechanism of wucai, we compared seedlings subjected to low-temperature and high-temperature treatments and found that the upregulated DEGs under LT were mainly related to photosynthesis. The results of the previous proteomic analysis of wucai WS-1 showed that the DEPs that respond to high and low temperatures are divided into several metabolites according to GO annotations: Redox homeostasis, photosynthesis, carbohydrate metabolism, heat shock proteins, signal transduction and metabolic processes. Many of these DEPs are classified as photosynthetic pathways, for example: PSBO1 (M4F7V3) PSBP1 (A0A078FRX3) PORC (M4EX79). This is similar to our research results [23].

### The growth of wucai under different temperature stress treatments based on physiological parameters

Related studies have shown that high temperature will lead to excessive growth or slow growth, and the relative water content of seedling leaves will also decrease after receiving temperature stress [25–27]. The plant height of aconite seedlings increased significantly under high temperature, which indicated that the wucai seedlings were excessive growing and the plants were not strong. The decrease of plant weight was mainly caused by the decrease of plant water content. Compared with the seedlings treated with high temperature, the seedlings treated with low temperature had stronger water retention ability of the leaves. Water is an important part of plant growth and metabolism, RWC show that maintain the situation of the plant body water content [28]. This study showed that high temperature leads to huge loss of moisture in wucai leaves, the main reason may be the high temperature results in the decrease of water absorption. Under high temperature stress, the reduction of RWC in leaves leads to stomatal closure, which leads to a decrease in CO_2_ utilization, which leads to a decrease in photosynthetic rate [29]. At low temperature, the relative water content of plants tends to decrease, leading to an increase in the concentration of cell fluid, thus improving the cold tolerance of plants. The RWC of plant leaves is an important indicator of plant tissue water status and reflects plant resistance. In this study, LT had a higher RWC than HT (Fig. 1).

### Evaluation of the degree of oxidative damage under temperature stress and the mechanism of improved antioxidant capacity

Temperature stress is one of the primary abiotic stresses experienced by plants during growth [30]. Under extreme temperature environments, a large amount of ROS, such as H_2_O_2_ and O_2_^.-^, is released in the seedlings, leading to membrane lipid peroxidation and increased membrane permeability, protein degradation, and loss of other cellular components [31, 32]. MDA is an important indicator for evaluating the degree of film oxidation, and the accumulation of ROS accelerates the lipid acylation of the cell membrane, increases the content of the toxic substance MDA, and destroys the integrity of the membrane structure [33]. MSI is used as the membrane stability coefficient to evaluate the integrity of the plasma membrane: the higher the MSI value, the better the integrity of the plasma membrane [34].

In this study, compared with the seedlings cultivated at room temperature, seedlings treated with LT or HT were enriched in H_2_O_2_, O_2_^.-^, and MDA following temperature stress, and the MSI decreased significantly, this is similar to the results of the Chakraborty’s study [35]. By comparing HT and LT, we found that the accumulation of H_2_O_2_, O_2_^−^, and MDA in the LT environment was significantly lower than that of HT, while MSI was higher in the former. This indicated that temperature stress will result in ROS accumulation in the seedlings and will cause a certain degree of membrane oxidative damage, but the degree of oxidative damage of the membrane lipids of LT was lower than that of HT (Fig. 2).

### The effects of different temperature stresses on the photosynthesis pathways in wucai

Photosynthesis is the first process affected by external temperature stress. We evaluated the changes in gas exchange parameters in the seedlings of wucai under different temperature stresses. Photosynthesis is very sensitive and easily affected by high temperature [36, 37], and low temperature will destroy many primary components of the photosynthetic machinery [38]. Under temperature stress, the P_N_ of the LT seedlings was higher than that of the HT seedlings, which indicated that the seedlings were more sensitive and susceptible to damage under high-temperature stress than under low-temperature stress. Temperature stress affects photosynthetic efficiency in two ways: stomatal restriction and non-stomatal restriction [39, 40]. When a decrease of P_N_ and Ci is accompanied by a decrease of Gs, the decrease of P_N_ is mainly due to stomatal restriction. In other cases, the decrease of P_N_ may be caused by non-stomatal factors, which is presumably due to the decrease in mesophyll cells under stress [41]. After 3 d of treatment with the two temperature stresses, g_s_ and E decreased significantly, whereas Ci increased significantly, indicating that the decrease in photosynthesis was caused by non-stomatal limitation. In previous studies, the loss of membrane integrity is related to plant tolerance to temperature. High temperature stress can affect membrane integrity, inhibit electron transfer in PSII and the level of key enzymes in Calvin cycle, and then affect carbon assimilation, resulting in an increase in the proportion of non-stomatal limitation [42, 43]. However, in our study, compared with HT, the MSI of wucai seedlings was higher in LT stress. Under LT conditions, wucai has higher stress resistance due to its more complete electron transport chain and higher activity of key enzymes in the Calvin cycle (Fig. 8).

PSII is the most sensitive component of the photosystem. Understanding PSII has been a key breakthrough in the alleviation of abiotic stress. According to reports, *PsbO* plays an important role in protecting PSII from light damage and stabilizing the function of the oxygen evolution complex (OEC) [44–47]. In higher plants, nonphotochemical chlorophyll fluorescence quenching is regulated by *PsbS*, thereby improving the plant’s own protective mechanism through the dissipation of excess light energy. In Głowacka’s research on *PsbS* overexpressed transgenic tobacco, it was found that the *PsbS* gene could reduce stomatal opening, reduce the transpiration rate, reduce water loss, and increase the plant RWC [48]. Fourier-transform infrared differential spectroscopy studies have confirmed that *PsbQ* interacts with *PsbP*. *PsbQ* can compensate for the damaged *PsbP* and cause a series of conformational changes in the Mn clusters of the water oxidation machinery [49]. Later studies on *PsbR* mutants of *Arabidopsis* found that *PsbP* and *PsbQ* were stably combined through *PsbR. PsbR* maintained the structural stability of PSII and enabled PSII to function [50, 51]. From the KEGG pathway analysis, we found that PsbP1 was upregulated in HT, while other *PSBPs* were downregulated, including *PsbO, PsbQ, PsbR, PsbS, PsbW, PsbY, Psb27,* and *Psb28*. In LT, no differential expression was observed in *PsbO* and *PsbP*, *PsbQ* was downregulated, and *Psb28* was upregulated. Based on previous studies, we suggest that the down-regulated expression of key PSII genes in the leaves of wucai under high temperature may be an important reason for the decreased PSII activity and limited electron transfer. On the contrary, low-temperature stress has little effect on the expression of key genes in the PSII response center, avoiding the photoinhibition of PSII in the leaves (Fig. 5).

Plant photosynthesis can be effectively measured using chlorophyll fluorescence. It can be used to further elucidate photosynthetic performance by reflecting the absorption, transmission, dissipation, and distribution of leaf light energy in the process of photosynthesis. Therefore, chlorophyll fluorescence analysis has also been widely used in the study of plant stress tolerance. The chlorophyll fluorescence parameter F_v_/F_m_ characterizes the conversion efficiency of light energy in the PSII reaction center. The results of this study show that the conversion efficiency of light energy was reduced in wucai under different temperature stresses, and LT had a higher F_v_/F_m_. Previous studies on *Arabidopsis* and other plants have also found that F_v_/F_m_ is highly correlated with low temperature tolerance [52–54]. Similarly, F_v_/F_o_ is also an important chlorophyll fluorescence parameter that reflects the activity of PSII and exhibits different response levels under different stresses. Our research showed that compared with the high temperature treatment, the F_v_/F_o_ of the seedlings decreased more slowly in the low temperature environment. The change in the relative variable fluorescence V_j_ reflects the electron transfer from QA to QB on the electron acceptor side of PSII. The change in V_j_ further indicates that the main reason for the decrease of PSII photochemical activity in the leaves of wucai under temperature stress was the PSII receptor. This is consistent with the results of previous research on *Medicago sativa* [55]. PI_abs_ reflects the capture of light energy in the PSII reaction center and the photosynthetic electron transfer ability between the two photosystems. The results showed that the PI_abs_ of wucai was reduced after temperature stress but was maintained at a higher level in LT. Based on the above, we speculate that the light conversion and performance of the wucai seedlings are affected by temperature stress, but they can maintain a better state in a low temperature environment, which is consistent with the transcriptome results. After absorbing light energy, one part of the light energy is transferred along the electron transport chain, and the other part is dissipated in the form of thermal energy. This protective mechanism prevents the reaction center from accumulating too much light energy and being inactivated or damaged [56]. ABS/RC represents the light energy absorbed per reaction center, TRO/RC represents the energy captured for QA reduction per reaction center, DIO/RC represents the energy dissipated per reaction center [57], and ETO/RC represents the energy used for electron transfer unit reaction center. After temperature stress, ABS/RC, DIO/RC, TRO/RC increased, while ETO/RC decreased, indicating that temperature stress inhibited electron transfer of PSII more than PSII, especially electron transfer from QA to QB. Temperature stress impedes the electron transfer of PSII, reduces the activity of reaction centers, and even leads to the inactivation of some reaction centers. As can be seen from our results, compared with the LT treatment, the ABS/RC, DIO/RC, TRO/RC of the HT treatment increased more, while the ETO/RC of the LT treatment decreased to a smaller extent. These results indicated that wucai had stronger reaction center activity under LT stress (Fig. 7).

The reaction center of PSI and PSII is the main location where ROS is produced. As an important signaling molecule, ROS can also directly cause cell damage by rapidly oxidizing cellular components, including lipids. A stable photosynthetic system is conducive to maintaining the dynamic balance of ROS in plants and the normal growth of plants under adversity. This is consistent with the results of the ROS-related indicators in this study.

### Analysis of the influence of different temperature stresses on the photosynthetic antenna protein pathway

*BrLhc cis*-acting elements were found to be involved in regulatory elements related to light responsiveness, *MYB* binding site, abscisic acid responsiveness, salicylic acid responsiveness, severe and stress responsiveness, low temperature responsiveness, and *MYBHv1* binding site. The *BrLhc* superfamily represents a class of antennae proteins, and *BrLhc* superfamily members not only participate in the process of light collection and transportation, but also participate in the regulation and distribution of excitation energy between PSI and PSII, the maintenance of thylakoid membrane structure, photoprotection, and the response to various stresses [58–60]. *MYB* TFs participate in the regulation of the cold stress response in rice and apples, and it has been indicated that *MYB* has a positive regulatory function on cold tolerance [7, 61, 62]. Abscisic acid plays an important role in maintaining the plant water balance and adaptation to adversity [63]. Previous studies on wheat, *Arabidopsis*, and potato have confirmed this [64, 65]. Zhang found that exogenous abscisic acid treatment of wheat enhanced the resistance of wheat in spring [66]. In previous studies on wheat, it was found that spraying exogenous salicylic acid enhanced the cold tolerance of plants by increasing the antioxidant capacity and expression of cold-responsive genes [67]. Studies in various plants have shown that Lhc gene expression is not only induced by light stress, but also by abiotic stress. For example, Capel and Jarillo found that low temperature treatment resulted in a rapid increase in the expression of *Lhcb1*, *3* at transcriptional level in *Arabidopsis* [68]. In this study, after low temperature treatment, all *BrLhc* genes in the photosynthetic antenna protein pathway were all up-regulated. Therefore, we speculated that the *BrLhc* genes was induced by low temperature, which is consistent with the results of previous studies. Both plants and algae can dissipate the excited state of chlorophyll into heat through a mechanism involving the Lhc protein that binds to lutein. According to reports, in addition to light capture, Lhc protein also participates in photoprotection through its lutein ligands. These lutein ligands are active in quenching chlorophyll leaflets and triplets and scavenging ROS [69]. Some recent studies have shown that photoinhibition take place in PSI at low temperatures and in the case of unbalanced linear electron transport chains [70–73]. PSI photoprotection is thought to be mainly mediated by oxygen scavenging enzymes [74], although recent evidence suggests that PSI antenna protein may play a related role in photoprotection against excess energy [75, 76], which can be regulated by carotenoids in the LHCI complex [77]. It seems logical to speculate that low temperature induces the high expression of *BrLhc* genes and participates in photoprotection, thereby improving the cold tolerance of wucai.

## Conclusion

This study compared the effects of different temperature stresses on the physiological characteristics of wucai seedlings. As a cold-resistant vegetable, wucai grew better under LT than under HT. The total soluble sugars, proline, and RWC were higher under LT than HT, indicating that wucai has better osmotic adjustment ability to resist temperature stress at a low temperature. We focused on the transcripts and pathways that LT specifically affects, specifically photosynthesis. By comparing the expression of DEGs in associated pathways and determining the related physiological indicators, we found that the photosynthetic performance of samples treated with LT was better than that of samples treated with HT. We speculate that the seedlings of wucai respond particularly to low temperature signals to stimulate the expression of genes in the photosynthesis pathway, as photosynthesis is an important plant biological process that provides energy for all life processes. In higher plants, the light-harvesting chlorophyll *a*/*b* binding (*Lhc*) protein plays a role in multiple processes that are essential for plant growth, development, and response to abiotic stress. *BrLhc* gene expression was upregulated under cold stress, which in turn affected the synthesis and metabolism of physiological and biochemical substances. The photosynthetic pathway can impact on the antioxidant capacity of plants. Under low temperature, wucai can improve its antioxidant capacity and alleviate the damage caused by stress. The regulation of the transcriptome confirms the unique protective mechanism of wucai under LT stress. The DEGs identified in this study can be used as targets for further verification and research to elucidate the unique temperature tolerance mechanism of wucai at the molecular level.

## Methods

### Plant materials, growth conditions, and treatments

‘WS-1’, a variety of non-heading Chinese cabbage with curled leaves, was developed by multigeneration self-pollination at Anhui Agricultural University. The seedlings were kept in a growth chamber at 25 ± 1 °C/18 ± 1 °C (day/night) with a 14/10 h light/dark photoperiod and approximately 320 μmol photons m^− 2^ s^− 1^. The seedlings were randomly divided into three groups once they had grown 4–5 true leaves. Each group was subjected to control (Cont, 25 ± 1 °C/18 ± 1 °C), low temperature (LT, 8 ± 1 °C/3 ± 1 °C), or high temperature (HT, 40 ± 1 °C/30 ± 1 °C) treatments. The experiment was repeated three times. All leaves samples were collected at the same point at 8:30 am after 72 h of treatment and immediately stored at − 80 °C for physiological analysis and RNA-Seq.

### Content of chlorophyll

The determination of Chl content was based on the method of Strain and Svec (1966) [78], with some modifications. Fresh leaf samples (0.2 g) were weighed and immersed in 25 mL extract solution containing acetone, ethanol, and water (volume ratio of 4.5: 4.5: 1) and treated in the dark for 24 h. The absorbance of the supernatant was then measured at 649 and 665 nm. The contents of Chl *a*, Chl *b*, and total Chl were calculated as follows:

$$ {\displaystyle \begin{array}{c}{\mathrm{C}}_{\mathrm{C}\mathrm{hl}\ \mathrm{a}}=13.95{\mathrm{A}}_{665}-6.88{\mathrm{A}}_{649},\\ {}{\mathrm{C}}_{\mathrm{C}\mathrm{hl}\ \mathrm{b}}=24.96{\mathrm{A}}_{649}-7.32{\mathrm{A}}_{665},\\ {}\mathrm{Total}\ \mathrm{Chl}=\mathrm{Chl}\ \mathrm{a}+\mathrm{Chl}\ \mathrm{b}.\end{array}} $$

### Measurement of Chl *a* fluorescence transience

Chlorophyll-*a* fluorescence emissions from the leaves were measured in vivo using a continuous-excitation fluorometer pocket plant efficiency analyzer (PEA, Hansatech, UK). Prior to measurement, the treated leaves were clamped using a leaf clamp (Hansatech) and dark-treated for 30 min. A total of 100,000 consecutive fluorescence trace measurements can be recorded every second, and the OJIP fluorescence induction curve of Chl can be completely measured in 1 s by a Poket PEA. The saturated red light emitted by the plant efficiency analyzer is 3000μmol•m^− 2^•s^− 1^ when measuring the fluorescence parameters of chlorophyll. Data were sampled at intervals of 10 μs in the first 300 μs. Fluorescence intensity was measured at 300 μs (F300μs), 2 ms (J point, FJ) and 30 ms (I point, Fi) 0.3–2 s (P point, FP). Chlorophyll *a* fluorescence transience was analyzed using the JIP-test formulae. The following data were also obtained: the initial fluorescence (Fo), maximum fluorescence (Fm), PSII potential photochemical activity (Fv/Fo), PSII maximum photochemical efficiency (Fv/Fm) at the inflection point (J), and relative variable fluorescence (Vj), and the performance index (PI) of absorption was calculated as PI (abs) = (RC/ABS) • [φPo/(1–φPo)] [ψo/(1–ψo)] [79]. The specific activity parameters of reaction center per unit of PSII, including ABS/RC, TRO/RC, ETO/RC, DIO/RC on the photosynthetic electron transport chain were directly provided by the instrument.

### Sample preparation, RNA extraction, and transcriptome sequencing

Total RNA was extracted from the Cont, HT, and LT leaf samples using a mirVana™ miRNA Isolation Kit Ambion-1561 according to the manufacturer’s instructions. RNA integrity was assessed with an Agilent 2100 Bioanalyzer (Agilent Technologies, Santa Clara, CA, USA). The mRNA was concentrated by Oligo (dT) attached magnetic beads. The mRNA was then broken into multiple fragments and used as a template to synthesize cDNA. The reference genome for this transcriptome sequencing comparison is the genome of the NCBI database: GCF_000309985.1 (Brapa_1.0). The purified cDNA was modified and selected for PCR amplification. Nine gene expression libraries were created and named Cont-1, Cont-2, Cont-3, HT-1, HT-2, HT-3, LT-1, LT-2, and LT-3 and sequenced by an Illumina HiSeqTM 2500, resulting in the generation of 125 bp or 150 bp double-ended reads.

### RNA-Seq, differential gene expression, and functional annotation

The transcriptome sequencing and analysis were completed by OE biotech Co., Ltd. (Shanghai, China). Raw reads were processed by Trimmomatic [80]. The reads containing ploy-N and the low quality reads were removed and the clean reads were obtained. Then the clean reads were mapped to reference genome by hisat2 [81]. Differential expression analysis was implemented by the DESeq R package to distinguish the DEGs between all treatment groups [82]. Significance was assessed used a negative binomial distribution test. The identification of false positives during the expression analysis of transcriptome sequencing is a major issue. Therefore, it is important to use the false discovery rate (FDR) error control method to perform multiple hypothesis test corrections on the *P*-value. The conditions for screening DEGs were *P* < 0.05 and log_2_|(foldchange)| > 1.

Enrichment analysis of DEGs was performed using Blast2GO software (Biobam, Valencia, Spain). A *P*-value of < 0.05 was considered as a condition of significant enrichment, and the algorithm of *P*-value determination was based on that of Pu et al. [83]. DEG enrichment in a Kyoto Encyclopedia of Genes and Genomes (KEGG) pathway was assessed by KOBAS software [84]. The basis on which the pathway was judged as significantly differentially expressed was a *P*-value < 0.05. Enriched KEGG pathways were ordered based on their *P*-value.

### Measurement of growth indicators

Growth parameter included plant height, single plant weight. Ten seedlings were randomly selected from each treatment group for measurement. Among them, plant height was measured by a ruler. Before measuring the weight of a single plant, the seedlings were washed and dried with deionized water first, and then weighed with an electronic balance. The relative water content (RWC) as a phenotypic parameter was determined according to Yuan et al. [85] and was calculated with the formula: RWC (%) = (fresh weight–dry weight)/ (turgor weight–dry weight) × 100%. After the shoots were washed with distilled water, their fresh weights were measured. The shoots were then floated on deionized water for 24 h in the dark, following which the turgor weight was recorded after wiping off excess water. The leaves were dried at 70 °C until their weights remained constant and were then used to determine the sample dry weight.

### Malondialdehyde (MDA), membrane stability index (MSI)

The MDA was measured in the wucai leaves according to Hu et al. [86]. The MDA calculation formula was as follows:

$$ \mathrm{MDA}=6.45\cdot \left({\mathrm{A}}_{532}-{\mathrm{A}}_{600}\right)-0.56\cdot {\mathrm{A}}_{450}\left(\mu \mathrm{mol}\cdot {\mathrm{L}}^{-1}\right). $$

The method of Abdelkrim et al. [87], was used to calculate the MSI as follows:

$$ \mathrm{MSI}=1-\left[\left(\mathrm{C}1\div \mathrm{C}2\right)\times 100\%\right]. $$

### Photosynthetic parameters

The P_N_, stomatal conductance (g_s_), intercellular CO_2_ concentration (Ci), transpiration rate (E), and various chlorophyll fluorescence parameters were simultaneously measured with a portable photosynthesis system (LI-6400, LI-COR Inc., Lincoln, NE, USA). The daily measurements were conducted between ZT2 (2 h after dawn) and ZT4 (4 h after dawn), and the third functional leaf samples of five biological replicate plants were tested. The measurement conditions were as follows: temperature, the measured temperature was the same as the treatment temperature of Cont, HT and LT [88]; RH, 70%; external CO_2_ concentration, 380 ± ws10 mol mol^− 1^; and light intensity, 1000 μmol photon m^− 2^ s^− 1^ [89].

### Bioinformatics analysis of the light-harvesting complex *(LHC)* family

To obtain the *BrLhc* family genes in wucai, the 34 known *LHC* genes published in *Arabidopsis thaliana* were downloaded from the TAIR genome database (https://www.arabidopsis.org/) and used as queries in a BLASTP search. The online website MEME (http://meme-suite.org/tools/meme) was used to predict the conserved motifs of the BrLhc protein sequence and construct a schematic diagram of the conserved motifs of the protein [90]. The structure of genes was visualized according to genome annotation files with TBtools [91]. MEGA7.0 software was used to compare the 47 BrLhc protein sequences of wucai, and the neighbor-joining method was used to construct a phylogenetic tree to analyze the evolutionary relationships between the *BrLhcs* of wucai [92]. The 2000 bp region upstream of the *BrLhc* genes was used as the promoter sequence by TBtools, and these sequences were submitted to Plant CARE (http://bioinformatics.psb.ugent.be/webtools/plantcare/html/) to predict the *BrLhc* promoter region *cis*-acting elements [93]. TBtools was used to visualize the promoter position.

### Gene expression

Total RNA was extracted using the RNA Extraction Kit (TaKaRa BIO, Japan) and then reverse transcribed into cDNA. Twelve transcripts were selected to validate the RNA-Seq analysis, and Primer Software version 5.0 was used to design gene-specific primers. *Actin* (*ACT*) was used as a reference gene to normalize the data. Please refer to Primer Table in this chapter (Suppl. Table S7). The quantitative real-time (qRT) PCR was performed using a Time System (Bio-Rad, California, USA) according to the manufacturer’s instructions. The gene expression data were analyzed using the 2^-ΔΔCt^ relative quantitative method in Excel (Microsoft Corp., Albuquerque, NM, USA).

### Statistical analysis

Data were expressed as the mean ± SD with three biological replicates. SPSS 22.0 (US SPSS Institute, Inc.) was used to analyze the differences, and using Duncan’s multiple range testat the *p* < 0.05 level of significance. The relational figures were drawn using GraphPad Prism v6.0 (http://www.graphpad.com/scientific-software/prism/).

## Supplementary Information

**Additional file 1.**

## Data Availability

The raw RNA-Seq data used in this study have been deposited in the Nation Center for Biotechnology Information (NCBI) Sequence Read Archive (SRA) database under the accession number PRJNA694542 (https://dataview.ncbi.nlm.nih.gov/object/PRJNA694542?reviewer=s0hddac5tgtoa3b9cfhhu7gr7c).

## Abbreviations

LT: Low temperature
HT: High temperature
Cont: Control
RWC: Relative water content
MDA: Malondialdehyde
MSI: Membrane stability index
Chl: Chlorophyll
DEGs: Differentially expressed genes
GO: Gene Ontology
KEGG: Kyoto Encyclopedia of Genes and Genomes
qRT-PCR: Quantitative real-time polymerase chain reaction
LHC: Light-harvesting chlorophyll protein complex
PSII: Photosystem II
PSI: Photosystem I
Fv/Fo: Efficiency of electron donation to PSII
Fv/Fm: Maximum quantum yield of PSII
ABS/RC: Absorption per reaction center at PSII/Ratio of active reaction centers in PSII
TRo/RC: Trapped energy flux per reaction center (t = 0)
ETo/RC: Electron transport flux per reaction center (t = 0)
DIo/RC: Dissipation energy flux per reaction center (t = 0)
PIabs: Performance index on absorption basis
V_j_: The relative fluorescence intensity of point J
