# Supplementary material for: Transcriptional profiling reveals changes in gene regulation and signaling transduction pathways during temperature stress in wucai (Brassica campestris L.)
Source: BMC Genomics. 2021 Sep 22;22:687. doi: 10.1186/s12864-021-07981-9 (PMC8456696; doi:10.1186/s12864-021-07981-9)
Supplement: Supplementary file 1 — Additional file 1. [file 12864_2021_7981_MOESM1_ESM.docx]

**Transcriptional profiling reveals changes in gene regulation and signaling transduction pathways during temperature stress in wucai (*Brassica campestris* L.)**

**Lingyun Yuan^1,2,3^** ^†^**, Yushan Zheng^1,2^** ^†^**, Libing Nie^1,2^, Liting Zhang^1,2^, Ying Wu^1,2^, Shidong Zhu^1,2,3^, Jinfeng Hou^1,2,3^,** **GuoLei Shan^1,2^, TongKun Liu^4^, Guohu Chen^1,2^, Xiaoyan Tang^1,2^ and Chenggang Wang^1,2,3, *^**

^1^ College of Horticulture, Vegetable Genetics and Breeding Laboratory, Anhui Agricultural University, 130 West Changjiang Road, 230036 Hefei, Anhui, China;

^2^ Provincial Engineering Laboratory for Horticultural Crop Breeding of Anhui, 130 West of Changjiang Road, 230036 Hefei, Anhui, China;

^3^ Wanjiang Vegetable Industrial Technology Institute, Maanshan, Anhui, 238200, China

^4^ Department of Horticulture, Nanjing Agricultural University, 210032 Nanjing, Jiangsu, China;

^†^ Lingyun Yuan and Yushan Zheng contributed equally to this work.

*Corresponding author: Chenggang Wang

Tel./Fax: +86 0551-65786212. E-mail: [cgwang@ahau.edu.cn](mailto:cgwang@ahau.edu.cn)


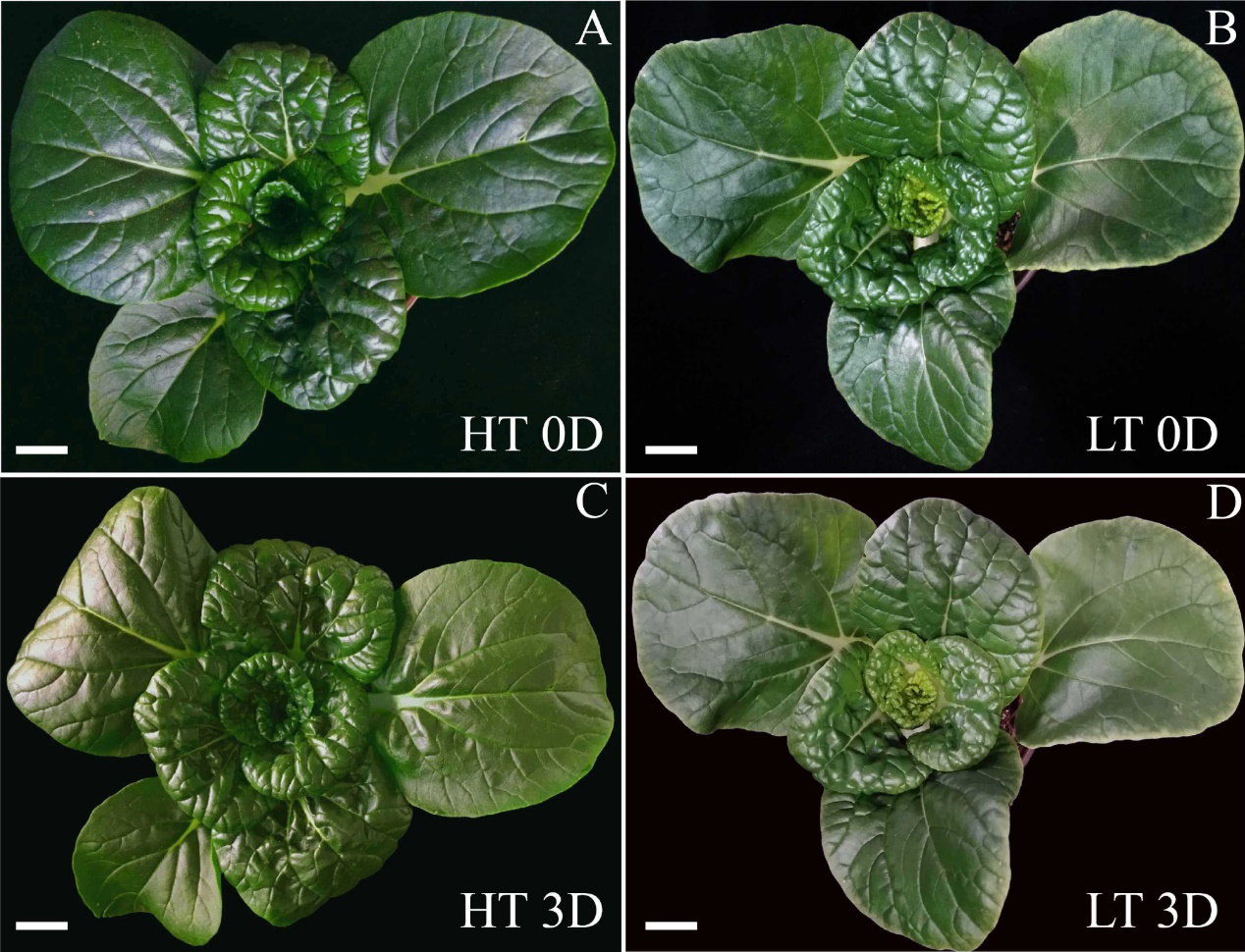


**Fig. S1** Wucai seedlings was treated at HT for 0d(a), at LT for 0d(b), at HT for 3d(c), and at LT for 3d(d). *bar*=2cm


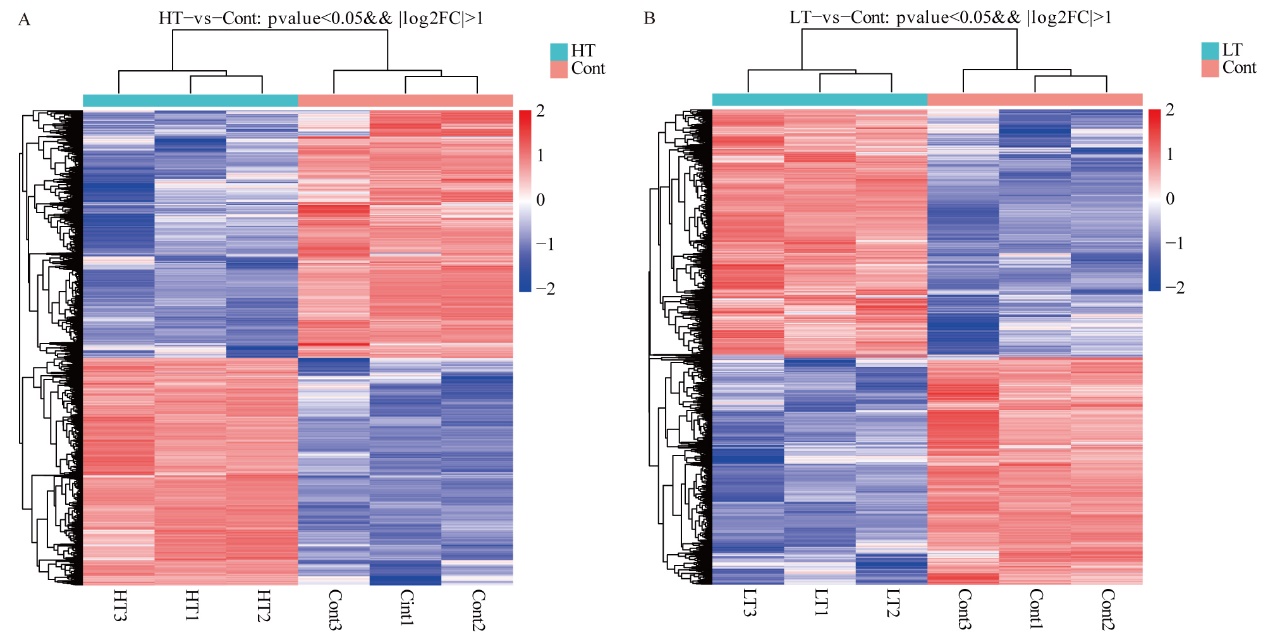


**Fig. S2** HT-vs-Cont-heatmap. (a) LT-vs-Cont-heatmap. (b) Hierarchical clustering of all of the DEGs was based on the log_10_RPKM values. The color spectrum from blue to red represents the gene expression intensity from low to high, respectively.


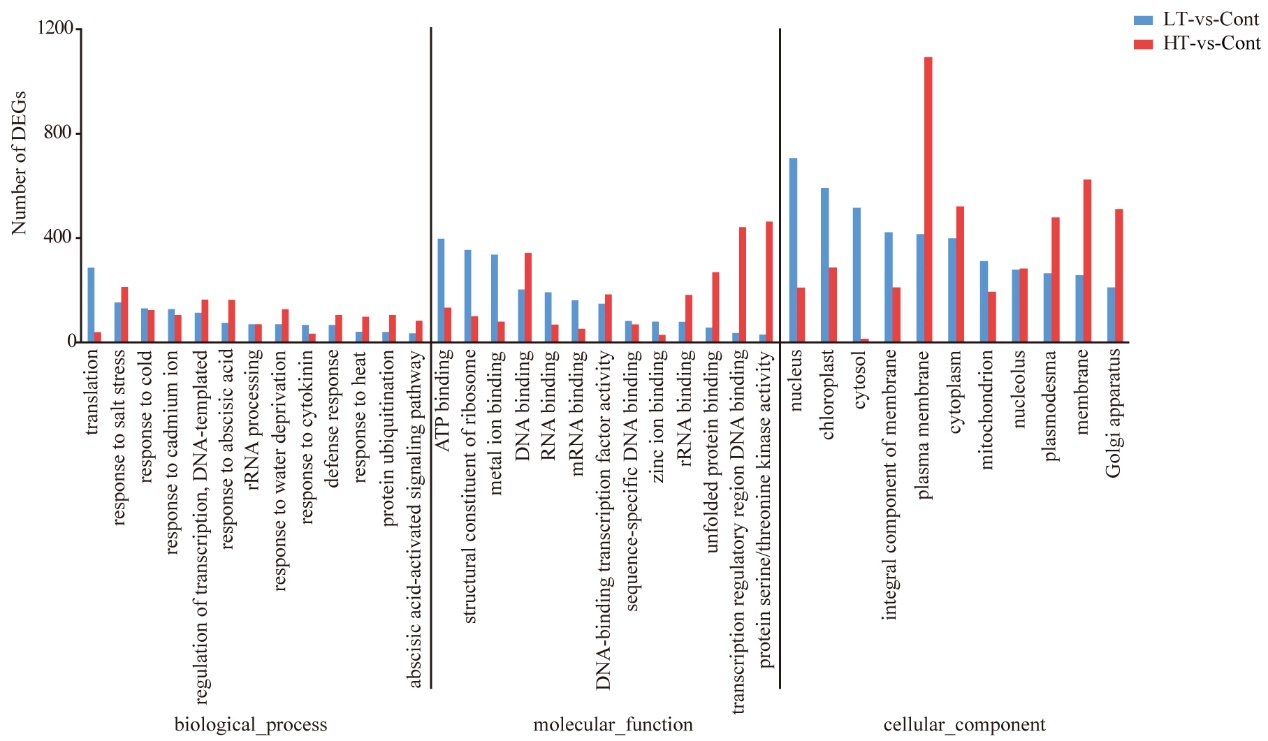


**Fig. S3** GO analyses of the DEGs in the LT-vs-Cont and HT-vs-Cont, the GO terms upregulated in the three GO categories.

**
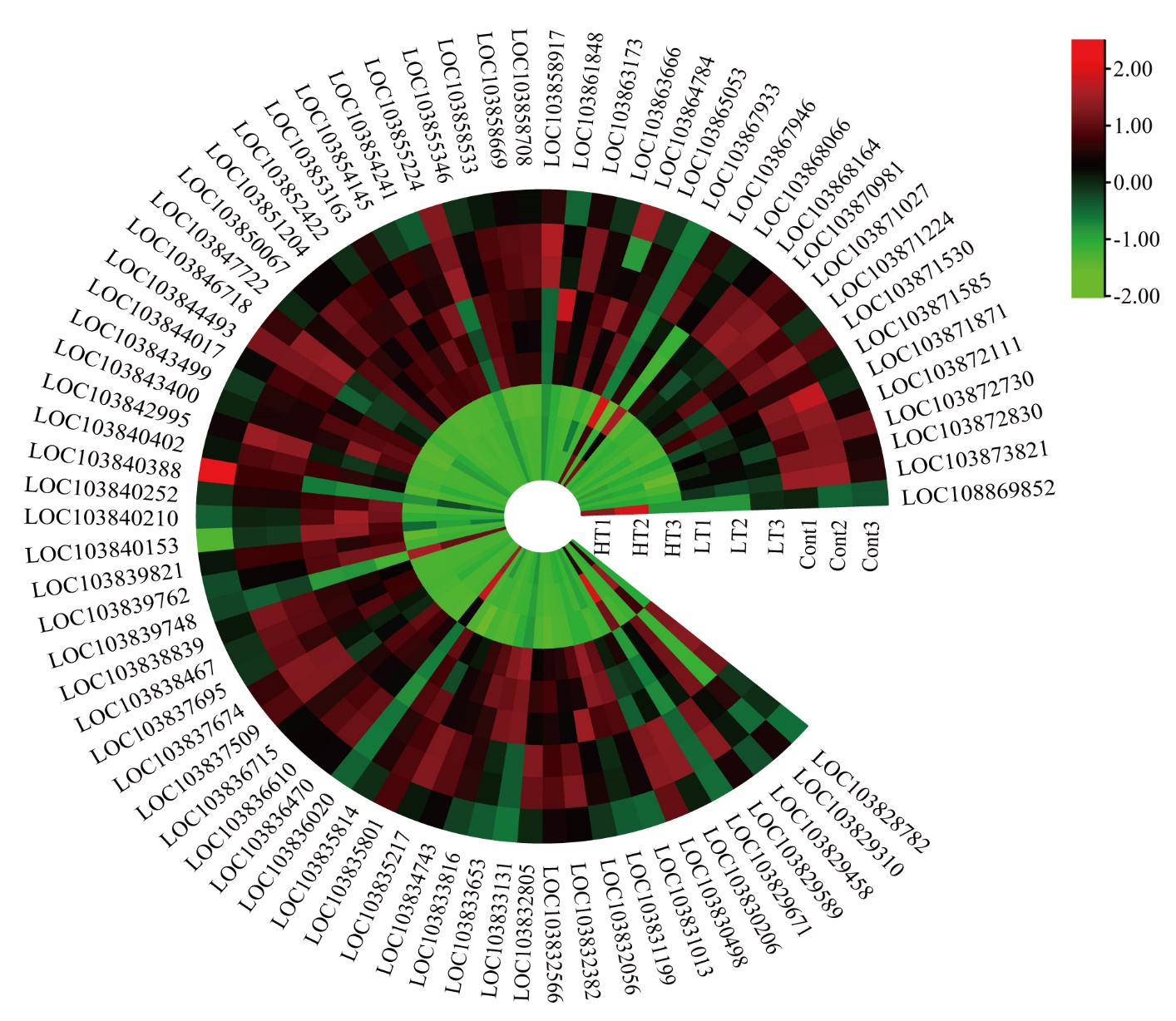
**

**Fig. S4** Heat map analysis of genes related to the photosynthetic pathway. The expression levels shown are based on FPKM data. The colored keys indicate the value of log2 (FPKM). Red represents high expression and green represents low expression. Each row represents a DEG.

**
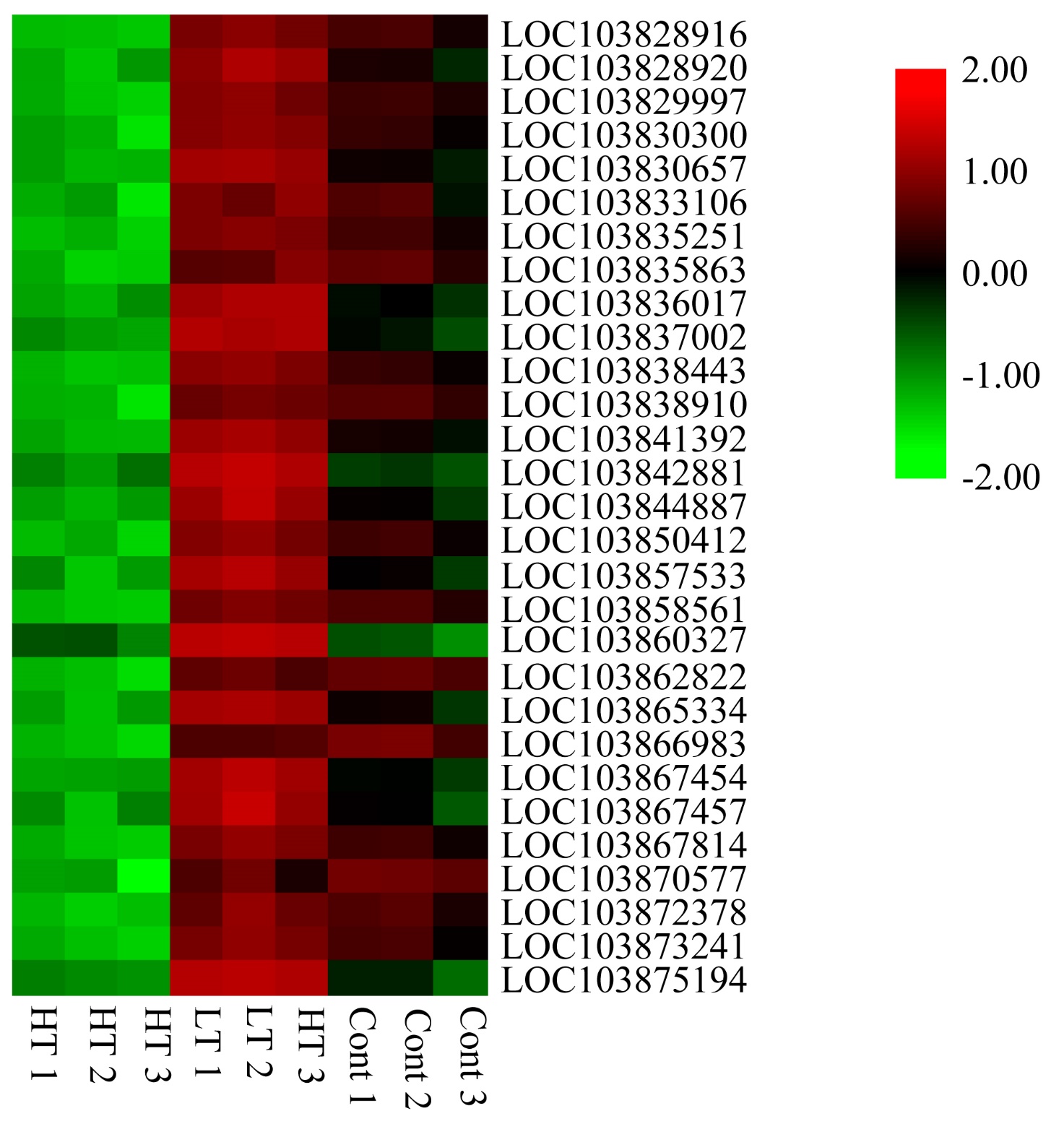
**

**Fig. S5** Heat map analysis of genes related to the photosynthetic antenna protein pathway. The expression levels shown are based on FPKM data. The colored keys indicate the value of log2 (FPKM). Red represents high expression and green represents low expression. Each row represents a DEG.


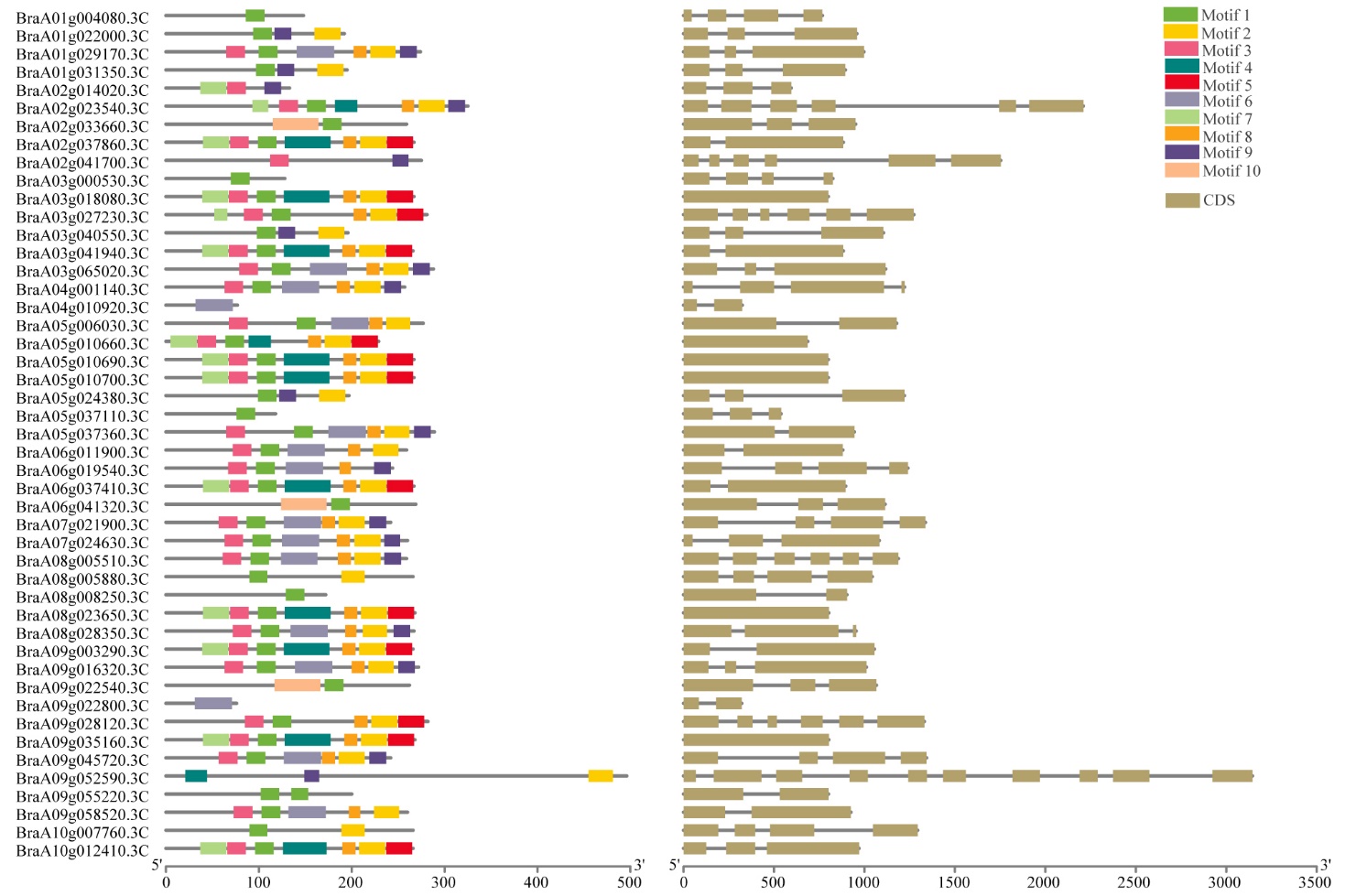


**Fig. S6** Distribution of conserved motifs among *BrLhc* superfamily proteins.

| Sample | Raw reads | Raw bases | Clean reads | Clean bases | Valid bases | Q30 | GC |
| --- | --- | --- | --- | --- | --- | --- | --- |
| HT1 | 44.11M | 6.62G | 43.30M | 6.04G | 91.21% | 94.54% | 46.97% |
| HT2 | 54.99M | 8.25G | 54.17M | 7.87G | 95.40% | 95.17% | 47.24% |
| HT3 | 47.03M | 7.06G | 46.20M | 6.45G | 91.44% | 94.35% | 47.08% |
| LT1 | 51.54M | 7.73G | 50.75M | 7.36G | 95.17% | 95.09% | 48.60% |
| LT2 | 47.42M | 7.11G | 46.61M | 6.63G | 93.19% | 94.73% | 48.70% |
| LT3 | 51.86M | 7.78G | 51.01M | 7.25G | 93.17% | 94.88% | 48.04% |
| Cont 1 | 50.37M | 7.56G | 49.55M | 6.97G | 92.30% | 94.64% | 47.66% |
| Cont 2 | 57.90M | 8.68G | 56.94M | 8.01G | 92.18% | 94.87% | 47.82% |
| Cont 3 | 46.37M | 6.96G | 45.54M | 6.39G | 91.88% | 94.47% | 47.57% |

**Table S1** Quality of RNA-seq data.

**Table S2** DEGs of HT-vs-Cont enriched in photosynthetic pathway

| Gene Id | Fold change | Up/Down | Gene symbol | Description |
| --- | --- | --- | --- | --- |
| LOC103829671 | 2.638655855 | Up | FD3 | ferredoxin, root R-B1 |
| LOC103836020 | 2.744976685 | Up | ATPC2 | ATP synthase gamma chain 2, chloroplastic |
| LOC103840153 | 2.193867952 | Up | FDC2 | ferredoxin-2-like |
| LOC103867933 | 11.60527698 | Up | PSBP1 | oxygen-evolving enhancer protein 2-1, chloroplastic-like |
| LOC103828782 | 0.02880407 | Down | PSAF | photosystem I reaction center subunit III, chloroplastic-like |
| LOC103829458 | 0.071603154 | Down | LFNR2 | ferredoxin--NADP reductase, leaf isozyme 2, chloroplastic |
| LOC103829589 | 0.071580612 | Down | PSBO1 | oxygen-evolving enhancer protein 1-1, chloroplastic |
| LOC103830206 | 0.031894639 | Down | FD2 | ferredoxin, leaf L-A |
| LOC103830498 | 0.235903355 | Down | PSBR | photosystem II 10 kDa polypeptide, chloroplastic |
| LOC103831013 | 0.189822506 | Down | PSBY | photosystem II core complex proteins psbY, chloroplastic-like |
| LOC103831199 | 0.099261572 | Down | PSBY | photosystem II core complex proteins psbY, chloroplastic |
| LOC103832056 | 0.196150511 | Down | PETE | plastocyanin |
| LOC103832382 | 0.077938939 | Down | PSBR | photosystem II 10 kDa polypeptide, chloroplastic-like |
| LOC103832566 | 0.13640133 | Down | PSAG | photosystem I reaction center subunit V, chloroplastic-like |
| LOC103832805 | 0.041758926 | Down | PSAH | photosystem I reaction center subunit VI, chloroplastic-like |
| LOC103833131 | 0.200334182 | Down | PSBS | photosystem II 22 kDa protein, chloroplastic |
| LOC103833653 | 0.285711123 | Down | FDC2 | ferredoxin-2-like |
| LOC103833816 | 0.095525827 | Down | PSAF | photosystem I reaction center subunit III, chloroplastic-like |
| LOC103834743 | 0.070829202 | Down | PSAE1 | photosystem I reaction center subunit IV A, chloroplastic-like |
| LOC103835217 | 0.048887218 | Down | PSAK | photosystem I reaction center subunit psaK, chloroplastic |
| LOC103835801 | 0.340458536 | Down | DRT112 | plastocyanin |
| LOC103835814 | 0.060477378 | Down | LFNR2 | ferredoxin--NADP reductase, leaf isozyme 2, chloroplastic |
| LOC103836470 | 0.095351523 | Down | PSBP | oxygen-evolving enhancer protein 2, chloroplastic |
| LOC103836610 | 0.079827339 | Down | PSB27-1 | photosystem II repair protein PSB27-H1, chloroplastic-like |
| LOC103836715 | 0.077804612 | Down | psaD1 | photosystem I reaction center subunit II-1, chloroplastic |
| LOC103837509 | 0.102760937 | Down | PSAN | photosystem I reaction center subunit N, chloroplastic-like |
| LOC103837674 | 0.112134289 | Down | LFNR1 | ferredoxin--NADP reductase, leaf isozyme 1, chloroplastic |
| LOC103837695 | 0.039488277 | Down | PSBO1 | oxygen-evolving enhancer protein 1-1, chloroplastic |
| LOC103838467 | 0.062878904 | Down | FD2 | ferredoxin, leaf L-A |
| LOC103838839 | 0.102228574 | Down | ATPD | ATP synthase subunit delta, chloroplastic |
| LOC103839762 | 0.061263793 | Down | PSBQ2 | oxygen-evolving enhancer protein 3-2, chloroplastic |
| LOC103839821 | 0.169017731 | Down | ATPC1 | ATP synthase gamma chain 1, chloroplastic-like |
| LOC103840210 | 0.043637629 | Down | PSAH1 | photosystem I reaction center subunit VI-1, chloroplastic |
| LOC103840252 | 0.068658147 | Down | PSAF | photosystem I reaction center subunit III, chloroplastic |
| LOC103840388 | 0.274176062 | Down | RFNR2 | ferredoxin--NADP reductase, root isozyme 2, chloroplastic |
| LOC103840402 | 0.085272655 | Down | PSAK | photosystem I reaction center subunit psaK, chloroplastic |
| LOC103842995 | 0.09966372 | Down | PNSL2 | photosynthetic NDH subunit of lumenal location 2, chloroplastic |
| LOC103843400 | 0.056651106 | Down | PSAO | photosystem I subunit O |
| LOC103843499 | 0.05117397 | Down | PSBP | oxygen-evolving enhancer protein 2, chloroplastic |
| LOC103844017 | 0.130385853 | Down | PSBP | oxygen-evolving enhancer protein 2, chloroplastic-like |
| LOC103844493 | 0.23734362 | Down | PSB27-1 | photosystem II repair protein PSB27-H1, chloroplastic |
| LOC103846718 | 0.158134931 | Down | petC | cytochrome b6-f complex iron-sulfur subunit, chloroplastic |
| LOC103847722 | 0.148900915 | Down | PSBS | photosystem II 22 kDa protein, chloroplastic |
| LOC103850067 | 0.077188416 | Down | PNSL3 | photosynthetic NDH subunit of lumenal location 3, chloroplastic-like |
| LOC103851204 | 0.125094863 | Down | ATPG | ATP synthase subunit b', chloroplastic |
| LOC103852422 | 0.061248753 | Down | PSBY | photosystem II core complex proteins psbY, chloroplastic-like |
| LOC103853163 | 0.161281884 | Down | PSBR | photosystem II 10 kDa polypeptide, chloroplastic-like |
| LOC103854145 | 0.103976287 | Down | PSAE1 | photosystem I reaction center subunit IV A, chloroplastic |
| LOC103854241 | 0.026605907 | Down | PSB28 | photosystem II reaction center PSB28 protein, chloroplastic |
| LOC103855224 | 0.040824469 | Down | PSAN | photosystem I reaction center subunit N, chloroplastic-like |
| LOC103855346 | 0.191308346 | Down | PSBO1 | oxygen-evolving enhancer protein 1-1, chloroplastic-like |
| LOC103858533 | 0.097378346 | Down | ATPD | ATP synthase subunit delta, chloroplastic-like |
| LOC103858669 | 0.142864674 | Down | ATPC1 | ATP synthase gamma chain 1, chloroplastic |
| LOC103858708 | 0.094563108 | Down | petC | cytochrome b6-f complex iron-sulfur subunit, chloroplastic |
| LOC103858917 | 0.058907063 | Down | PSBQ2 | oxygen-evolving enhancer protein 3-1, chloroplastic |
| LOC103861848 | 0.136865713 | Down | PSB28 | photosystem II reaction center PSB28 protein, chloroplastic-like |
| LOC103863173 | 0.088085761 | Down | PPL1 | psbP-like protein 1, chloroplastic |
| LOC103863666 | 0.2001235 | Down | PSAL | photosystem I reaction center subunit XI, chloroplastic |
| LOC103864784 | 0.475392383 | Down | FD3 | ferredoxin, root R-B2 |
| LOC103865053 | 0.135987486 | Down | PSBW | photosystem II reaction center W protein, chloroplastic |
| LOC103867946 | 0.182595618 | Down | PSBW | photosystem II reaction center W protein, chloroplastic-like |
| LOC103868164 | 0.127646723 | Down | ATPC1 | ATP synthase gamma chain 1, chloroplastic-like |
| LOC103869852 | 0.081281722 | Down | PSAH1 | photosystem I reaction center subunit VI-1, chloroplastic |
| LOC103870981 | 0.163475484 | Down | PNSL3 | photosynthetic NDH subunit of lumenal location 3, chloroplastic |
| LOC103871027 | 0.02601797 | Down | PSAG | photosystem I reaction center subunit V, chloroplastic-like |
| LOC103871224 | 0.048452972 | Down | PSAH | photosystem I reaction center subunit VI, chloroplastic-like |
| LOC103871530 | 0.187463359 | Down | FDC1 | ferredoxin-1 |
| LOC103871585 | 0.05465551 | Down | PSAO | photosystem I subunit O |
| LOC103871871 | 0.106607189 | Down | FD1 | ferredoxin-1, chloroplastic |
| LOC103872111 | 0.041965417 | Down | PNSL2 | photosynthetic NDH subunit of lumenal location 2, chloroplastic-like |
| LOC103872730 | 0.348512671 | Down | PSBO2 | oxygen-evolving enhancer protein 1-2, chloroplastic |
| LOC103872830 | 0 | Down | DRT112 | plastocyanin |
| LOC103873821 | 0.079618492 | Down | PSAN | photosystem I reaction center subunit N, chloroplastic |
| LOC103829310 | 0.326157078 | Down | ATPD | ATP synthase subunit delta, chloroplastic-like |
| LOC103868066 | 0.384831744 | Down | FD1 | ferredoxin-1, chloroplastic |

**Table S3** DEGs of LT-vs-Cont enriched in photosynthetic pathway

| Gene Id | Fold change | Up/Down | Gene symbol | Description |
| --- | --- | --- | --- | --- |
| LOC103828782 | 3.174161535 | Up | PSAF | photosystem I reaction center subunit III, chloroplastic-like |
| LOC103829458 | 2.051434576 | Up | LFNR2 | ferredoxin--NADP reductase, leaf isozyme 2, chloroplastic |
| LOC103831199 | 2.739640434 | Up | PSBY | photosystem II core complex proteins psbY, chloroplastic |
| LOC103833131 | 4.853472154 | Up | PSBS | photosystem II 22 kDa protein, chloroplastic |
| LOC103833653 | 2.00623323 | Up | FDC2 | ferredoxin-2-like |
| LOC103840153 | 2.193867952 | Up | FDC2 | ferredoxin-2-like |
| LOC103840210 | 2.649864616 | Up | PSAH1 | photosystem I reaction center subunit VI-1, chloroplastic |
| LOC103861848 | 2.08971487 | Up | PSB28 | photosystem II reaction center PSB28 protein, chloroplastic-like |
| LOC103829310 | 0.326157078 | Down | ATPD | ATP synthase subunit delta, chloroplastic-like |
| LOC103840388 | 0.320519277 | Down | RFNR2 | ferredoxin--NADP reductase, root isozyme 2, chloroplastic |
| LOC103855224 | 0.276968876 | Down | PSAN | photosystem I reaction center subunit N, chloroplastic-like |
| LOC103858917 | 0.24550649 | Down | PSBQ2 | oxygen-evolving enhancer protein 3-1, chloroplastic |
| LOC103868066 | 0.384831744 | Down | FD1 | ferredoxin-1, chloroplastic |

**Table S4** DEGs of HT-vs-Cont enriched in photosynthetic antenna protein pathway

| Gene Id | Fold change | Up/Down | Gene symbol | Description |
| --- | --- | --- | --- | --- |
| LOC103828916 | 0.013977582 | Down | LHCB1.3 | chlorophyll a-b binding protein 1, chloroplastic |
| LOC103828920 | 0.062190907 | Down | LHCB1.3 | chlorophyll a-b binding protein 1, chloroplastic-like |
| LOC103829997 | 0.064625758 | Down | LHCA1 | chlorophyll a-b binding protein 6, chloroplastic |
| LOC103830300 | 0.159719455 | Down | LHCA2 | photosystem I chlorophyll a/b-binding protein 2, chloroplastic |
| LOC103830657 | 0.169920117 | Down | LHCA3 | photosystem I chlorophyll a/b-binding protein 3-1, chloroplastic |
| LOC103833106 | 0.104812583 | Down | LHCA5 | photosystem I chlorophyll a/b-binding protein 5, chloroplastic |
| LOC103835251 | 0.018742175 | Down | LHCB1.2 | chlorophyll a-b binding protein 2, chloroplastic-like |
| LOC103835863 | 0.122686954 | Down | LHCA6 | photosystem I chlorophyll a/b-binding protein 6, chloroplastic |
| LOC103836017 | 0.131382765 | Down | CAP10B | chlorophyll a-b binding protein CP24 10B, chloroplastic-like |
| LOC103837002 | 0.191916627 | Down | LHCB2.4 | chlorophyll a-b binding protein 36, chloroplastic |
| LOC103838443 | 0.032365074 | Down | LHCA3 | photosystem I chlorophyll a/b-binding protein 3-1, chloroplastic |
| LOC103838910 | 0.083211478 | Down | LHCB5 | chlorophyll a-b binding protein CP26, chloroplastic-like |
| LOC103841392 | 0.066557781 | Down | LHCA1 | chlorophyll a-b binding protein 6, chloroplastic |
| LOC103842881 | 0.364262336 | Down | CAP10B | chlorophyll a-b binding protein CP24, chloroplastic-like |
| LOC103844887 | 0.080177324 | Down | LHCB3 | chlorophyll a-b binding protein 3, chloroplastic |
| LOC103850412 | 0.068879791 | Down | LHCB4.1 | chlorophyll a-b binding protein CP29.1, chloroplastic |
| LOC103857533 | 0.164424015 | Down | CAB1 | chlorophyll a-b binding protein 1, chloroplastic |
| LOC103858561 | 0.035137284 | Down | LHCB5 | chlorophyll a-b binding protein CP26, chloroplastic |
| LOC103862822 | 0.098790178 | Down | LHCA2 | photosystem I chlorophyll a/b-binding protein 2, chloroplastic-like |
| LOC103865334 | 0.07545179 | Down | CAB1 | chlorophyll a-b binding protein 1, chloroplastic-like |
| LOC103866983 | 0.042088459 | Down | LHCB4.3 | chlorophyll a-b binding protein CP29.3, chloroplastic |
| LOC103867454 | 0.110579018 | Down | CAB1 | chlorophyll a-b binding protein 1, chloroplastic-like |
| LOC103867457 | 0.190482354 | Down | CAB1 | chlorophyll a-b binding protein 1, chloroplastic-like |
| LOC103867814 | 0.021543409 | Down | LHCB1.3 | uncharacterized LOC103867814 |
| LOC103870577 | 0.155880215 | Down | LHCB4.2 | chlorophyll a-b binding protein CP29.2, chloroplastic |
| LOC103872378 | 0.056150778 | Down | CAP10B | chlorophyll a-b binding protein CP24, chloroplastic |
| LOC103873241 | 0.077527864 | Down | LHCA4 | chlorophyll a-b binding protein 4, chloroplastic |
| LOC103875194 | 0.259271338 | Down | LHCB2.4 | chlorophyll a-b binding protein 5, chloroplastic-like |

**Table S5** DEGs of LT-vs-Cont enriched in photosynthetic antenna protein pathway

| Gene Id | Fold change | Up/Down | Gene symbol | Description |
| --- | --- | --- | --- | --- |
| LOC103828916 | 3.344094279 | Up | LHCB1.3 | chlorophyll a-b binding protein 1, chloroplastic |
| LOC103828920 | 7.941878006 | Up | LHCB1.3 | chlorophyll a-b binding protein 1, chloroplastic-like |
| LOC103829997 | 2.485324766 | Up | LHCA1 | chlorophyll a-b binding protein 6, chloroplastic |
| LOC103830300 | 2.249973156 | Up | LHCA2 | photosystem I chlorophyll a/b-binding protein 2, chloroplastic |
| LOC103830657 | 4.432547407 | Up | LHCA3 | photosystem I chlorophyll a/b-binding protein 3-1, chloroplastic |
| LOC103835251 | 3.696042512 | Up | LHCB1.2 | chlorophyll a-b binding protein 2, chloroplastic-like |
| LOC103836017 | 9.838732843 | Up | CAP10B | chlorophyll a-b binding protein CP24 10B, chloroplastic-like |
| LOC103837002 | 11.89540519 | Up | LHCB2.4 | chlorophyll a-b binding protein 36, chloroplastic |
| LOC103838443 | 4.125420181 | Up | LHCA3 | photosystem I chlorophyll a/b-binding protein 3-1, chloroplastic |
| LOC103841392 | 7.256052395 | Up | LHCA1 | chlorophyll a-b binding protein 6, chloroplastic |
| LOC103842881 | 19.33907961 | Up | CAP10B | chlorophyll a-b binding protein CP24, chloroplastic-like |
| LOC103844887 | 15.15239734 | Up | LHCB3 | chlorophyll a-b binding protein 3, chloroplastic |
| LOC103850412 | 2.777441641 | Up | LHCB4.1 | chlorophyll a-b binding protein CP29.1, chloroplastic |
| LOC103857533 | 7.512488527 | Up | CAB1 | chlorophyll a-b binding protein 1, chloroplastic |
| LOC103858561 | 2.049374779 | Up | LHCB5 | chlorophyll a-b binding protein CP26, chloroplastic |
| LOC103860327 | 50.98646109 | Up | LHCB2.1 | chlorophyll a-b binding protein 151, chloroplastic |
| LOC103865334 | 12.05928326 | Up | CAB1 | chlorophyll a-b binding protein 1, chloroplastic-like |
| LOC103867454 | 14.05962473 | Up | CAB1 | chlorophyll a-b binding protein 1, chloroplastic-like |
| LOC103867457 | 8.992856737 | Up | CAB1 | chlorophyll a-b binding protein 1, chloroplastic-like |
| LOC103867814 | 3.489021918 | Up | LHCB1.3 | uncharacterized LOC103867814 |
| LOC103873241 | 2.253574036 | Up | LHCA4 | chlorophyll a-b binding protein 4, chloroplastic |
| LOC103875194 | 19.38943421 | Up | LHCB2.4 | chlorophyll a-b binding protein 5, chloroplastic-like |

**Table S6** Analysis of genomic information and protein characterization of *BrLHC* gene family members in Wucai

| Subfamily | Gene ID | Gene name | Chromosome | Position（5'→3'） | ORF/(bp) | AA length/(aa) | pI | Molecular weight/(kDa) | Subcellular localization |  |
| --- | --- | --- | --- | --- | --- | --- | --- | --- | --- | --- |
|  |  |  |  |  |  |  |  |  |  |  |
| LHCA | BraA01g004080.3C | Lhca3 | A01 | 1980357-1981124(+) | 447 | 148 | 11.48 | 14821.06 | chlo |  |
|  | BraA01g029170.3C | Lhca3.1 | A01 | 19265135-19266132(+) | 825 | 274 | 7.85 | 29258.55 | chlo |  |
|  | BraA03g065020.3C | Lhca3.2 | A03 | 37483096-37484213(-) | 867 | 288 | 6.6 | 31822.4 | chlo |  |
|  | BraA04g001140.3C | Lhca2.2 | A04 | 741827-743049(-) | 774 | 257 | 6.9 | 27605.77 | chlo |  |
|  | BraA07g021900.3C | Lhca1 | A07 | 17768383-17769720(-) | 729 | 242 | 6.22 | 26040.01 | vacu |  |
|  | BraA07g024630.3C | Lhca2 | A07 | 19251467-19252549(+) | 783 | 260 | 6.42 | 27907.06 | chlo |  |
|  | BraA08g005510.3C | Lhca5 | A08 | 4315749-4316936(-) | 780 | 259 | 7.95 | 27910.21 | chlo |  |
|  | BraA08g028350.3C | Lhca2.1 | A08 | 19820533-19821487(-) | 804 | 267 | 5.14 | 29368.49 | chlo |  |
|  | BraA09g016320.3C | Lhca3.3 | A09 | 10025155-10026166(-) | 819 | 272 | 7.86 | 29113.39 | chlo |  |
|  | BraA09g045720.3C | Lhca1.1 | A09 | 34899148-34900491(-) | 729 | 242 | 6.22 | 26042.03 | vacu |  |
| LHCB | BraA02g014020.3C | Lhcb3.1 | A02 | 7186122-7186717(-) | 402 | 133 | 4.9 | 14262.02 | chlo |  |
|  | BraA02g023540.3C | Lhcb7 | A02 | 13811793-13814003(+) | 978 | 325 | 8.31 | 36113.77 | plas |  |
|  | BraA02g037860.3C | Lhcb2.4.1 | A02 | 26487364-26488247(+) | 804 | 267 | 5.62 | 28666.54 | chlo |  |
|  | BraA02g041700.3C | Lhcb4.1.1 | A02 | 28908885-28910638(+) | 828 | 275 | 8.93 | 30935.87 | chlo |  |
|  | BraA03g018080.3C | Lhcb1.4.1 | A03 | 8434314-8435114(-) | 804 | 267 | 5.17 | 28232.16 | chlo |  |
|  | BraA03g027230.3C | Lhcb5.2 | A03 | 13465446-13466719(+) | 846 | 281 | 6.34 | 30051.59 | chlo |  |
|  | BraA03g041940.3C | Lhcb2 | A03 | 21055368-21056251(-) | 801 | 266 | 5.47 | 28603.54 | chlo |  |
|  | BraA04g010920.3C | Lhcb4.2.3 | A04 | 8648397-8648721(-） | 234 | 77 | 5.08 | 8658.92 | cyto |  |
|  | BraA05g006030.3C | Lhcb4.3 | A05 | 3038140-3039318（-） | 834 | 277 | 5.24 | 30101.46 | plas |  |
|  | BraA05g010660.3C | Lhcb1.4.3 | A05 | 5707059-5707745（-） | 690 | 229 | 6.97 | 24862.37 | chlo |  |
|  | BraA05g010690.3C | Lhcb1.4.4 | A05 | 5718804-5719604（-） | 804 | 267 | 5.16 | 28154.09 | chlo |  |
|  | BraA05g010700.3C | Lhcb1.4.5 | A05 | 5720221-5721021(+) | 804 | 267 | 5.16 | 28168.12 | chlo |  |
|  | BraA05g037360.3C | Lhcb4.2.2 | A05 | 25779387-25780331（-) | 870 | 289 | 5.64 | 31114.36 | chlo |  |
|  | BraA06g011900.3C | Lhcb6.2 | A06 | 6431137-6432017(-) | 780 | 259 | 8.8 | 27497.5 | chlo |  |
|  | BraA06g019540.3C | Lhcb4 | A06 | 11205894-11207134(+) | 735 | 244 | 6.1 | 26832.47 | chlo |  |
|  | BraA06g037410.3C | Lhcb2.4.2 | A06 | 24987426-24988322(-) | 804 | 267 | 5.62 | 28666.54 | chlo |  |
|  | BraA08g023650.3C | Lhcb2.4.3 | A08 | 17431324-17432127(-) | 807 | 268 | 5.32 | 28296.29 | chlo |  |
|  | BraA09g003290.3C | Lhcb2.4.4 | A09 | 2050601-2051656(+) | 801 | 266 | 5.62 | 28609.49 | chlo |  |
|  | BraA09g022800.3C | Lhcb4.2.1 | A09 | 15086211-15086532(-) | 231 | 76 | 9.74 | 8427.84 | chlo |  |
|  | BraA09g028120.3C | Lhcb5.1 | A09 | 19217166-19218497(-) | 849 | 282 | 6.34 | 30261.93 | chlo |  |
|  | BraA09g035160.3C | Lhcb1.4.2 | A09 | 27830055-27830858(+) | 807 | 268 | 5.33 | 28326.32 | chlo |  |
|  | BraA09g058520.3C | Lhcb6.1 | A09 | 41458435-41459360(+) | 783 | 260 | 8.78 | 27734.8 | chlo |  |
|  | BraA10g012410.3C | Lhcb3.2 | A10 | 10248504-10249474(+) | 801 | 266 | 4.96 | 28698.77 | chlo |  |
| ELIP | BraA01g022000.3C | ELIP4 | A01 | 12195553-12196511(-) | 579 | 192 | 11.48 | 14821.06 | chlo |  |
|  | BraA01g031350.3C | ELIP2 | A01 | 21215612-21216506(+) | 588 | 195 | 9.55 | 20335.6 | chlo |  |
|  | BraA03g040550.3C | ELIP3 | A03 | 20183468-20184573(-) | 591 | 196 | 9.24 | 20490.83 | chlo |  |
|  | BraA05g024380.3C | ELIP1 | A05 | 18231358-18232579(+) | 594 | 197 | 9.61 | 20715 | chlo |  |
| OHP | BraA03g000530.3C | OHP1.1 | A03 | 231998-232823(+) | 387 | 128 | 9.6 | 13742.13 | chlo |  |
|  | BraA05g037110.3C | OHP1.2 | A05 | 25672985-25673524(-) | 357 | 118 | 10.15 | 12673.16 | extr |  |
|  | BraA08g008250.3C | OHP2.1 | A08 | 7238389-7239292(+) | 519 | 172 | 9.44 | 18581.41 | chlo |  |
| SEP | BraA09g055220.3C | SEP2 | A09 | 39875896-39876697(+) | 603 | 200 | 4.85 | 21527.56 | chlo |  |
| Lil | BraA09g022540.3C | Lil3 | A09 | 14946652-14947718(-) | 789 | 262 | 5.15 | 28835.77 | chlo |  |
|  | BraA06g041320.3C | Lil3.2 | A06 | 27266583-27267697(+) | 810 | 269 | 4.9 | 29566.54 | chlo |  |
|  | BraA02g033660.3C | Lil3.1 | A02 | 23396381-23397332(-) | 780 | 259 | 4.88 | 28792.48 | chlo |  |
| PSBS | BraA08g005880.3C | PSBS1 | A08 | 4672893-4673936(-) | 801 | 266 | 9.25 | 27992.83 | chlo |  |
|  | BraA10g007760.3C | PSBS2 | A10 | 5106133-5107427(+) | 801 | 266 | 9.4 | 28187.07 | chlo |  |
| FC | BraA09g052590.3C | FC II | A09 | 38364696-38367841(-) | 1491 | 496 | 5.27 | 54928.93 | chlo |  |

**Table S7** Primer sequences used for qRT-PCR

| Use | Primer name | Forward primer (5'- 3') | Reverse primer (5'- 3') |
| --- | --- | --- | --- |
| qRT-PCR | LOC103865334 | GAGTTCCCAGGAGACTACGG | AAACGGCTTCTCCGAACTTG |
|  | LOC103867457 | CGCAGAGTTGAAGGTGAAGG | CAGCGAGATTCTCCAGAGGT |
|  | LOC103842881 | GGCTTGGTTCTTCGTTCCTC | AAAGCCGGGTCCTTTCCTAA |
|  | LOC103841392 | CTCCAAGGACCCGAAGAAGT | CCAGGTTCTCCAATGGTCCT |
|  | LOC103835251 | GCAGCATCAGAAGTCCTTGG | GTCCCATCCGTAGTCTCCTG |
|  | LOC103865334 | GAGTTCCCAGGAGACTACGG | AAACGGCTTCTCCGAACTTG |
|  | LOC103828920 | GAGTTCCCAGGAGACTACGG | AAGATCTGTGAGCCAGCCTT |
|  | LOC103838443 | GTTGCAGGACTGGTACAACC | TAGGATTGCGAGCATAGCCA |
|  | LOC103830657 | ACTTGGATGGCAGCTTACCT | GCAGTGTCTGCAGGAATCAG |
|  | LOC103858501 | CTCTTAGCTAGCGACCCACA | TTAGGTTGATCCGCTCGACA |
|  | LOC103856040 | TCGTTGGACAGGCTCTCTTT | TCCCACTTCCCTCAAGTGTC |
|  | LOC103828916 | GGAGGACTCGACTACTTGGG | TAACCTTCAACGGCTCCCAT |
